# Linking the evolution of development of stem vascular system in Nyctaginaceae and its correlation to habit and species diversification

**DOI:** 10.1186/s13227-021-00190-1

**Published:** 2022-01-29

**Authors:** Israel L. Cunha Neto, Marcelo R. Pace, Rebeca Hernández-Gutiérrez, Veronica Angyalossy

**Affiliations:** 1 Laboratório de Anatomia Vegetal, Departamento de Botânica, Instituto de Biociências, Universidade de São Paulo, Rua do Matão 277, São Paulo, SP Brazil; 2 School of Integrative Plant Sciences and L.H. Bailey Hortorium, Cornell University, Ithaca, NY 14853 USA; 3 Departamento de Botánica, Instituto de Biología, Universidad Nacional Autónoma de México, Ciudad Universitaria, Circuito Zona Deportiva s/n, Ciudad Universitaria, 04510 Coyoacán, Mexico City, Mexico

**Keywords:** Anatomy, BAMM, Caryophyllales, Continuum morphology, Developmental processes, Evolution, Ontogeny, Vascular tissues

## Abstract

**Background:**

Alternative patterns of secondary growth in stems of Nyctaginaceae is present in all growth habits of the family and have been known for a long time. However, the interpretation of types of cambial variants have been controversial, given that different authors have given them different developmental interpretations. The different growth habits coupled with an enormous stem anatomical diversity offers the unique opportunity to investigate the evolution of complex developments, to address how these anatomies shifted within habits, and how the acquisition of novel cambial variants and habit transitions impacted the diversification of the family.

**Methods:**

We integrated developmental data with a phylogenetic framework to investigate the diversity and evolution of stem anatomy in Nyctaginaceae using phylogenetic comparative methods, reconstructing ancestral states, and examining whether anatomical shifts correspond to species diversification rate shifts in the family.

**Results:**

Two types of cambial variants, interxylary phloem and successive cambia, were recorded in Nyctaginaceae, which result from four different ontogenies. These ontogenetic trajectories depart from two distinct primary vascular structures (regular or polycyclic eustele) yet, they contain shared developmental stages which generate stem morphologies with deconstructed boundaries of morphological categories (continuum morphology). Unlike our a priori hypotheses, interxylary phloem is reconstructed as the ancestral character for the family, with three ontogenies characterized as successive cambia evolving in few taxa. Cambial variants are not contingent on habits, and their transitions are independent from species diversification.

**Conclusions:**

Our findings suggest that multiple developmental mechanisms, such as heterochrony and heterotopy, generate the transitions between interxylary phloem and successive cambia. Intermediate between these two extremes are present in Nyctaginaceae, suggesting a continuum morphology across the family as a generator of anatomical diversity.

**Supplementary Information:**

The online version contains supplementary material available at 10.1186/s13227-021-00190-1.

## Background

In the context of evolutionary developmental biology and phylogenetic research, morphological and anatomical comparative studies have played a major role in unravelling the complexity and diversity of both living and fossil organisms [1, 2]. One of the fundamental pillars of this discipline is to investigate how developmental modifications contribute to the diversity of phenotypes in nature [1, 3–5]. In stem vascular development, previous studies have demonstrated different developmental programmes interacting in the evolution of various anatomical architectures, which are linked to hydraulic and biomechanical functions critical to water and sugar transport, storage, sustain and flexibility [6–9]. Therefore, by studying the developmental patterns that generate the diversity of stem vascular systems within a phylogenetic context, we can learn about the evolutionary mechanisms that have shaped the evolution of plant stems.

Secondary growth derived from a circular bifacial vascular cambium producing both wood (secondary xylem) and inner bark (secondary phloem), i.e., regular secondary growth, is thought to have originated in the Carboniferous 330 million years ago in the common ancestor of progymnosperms, gymnosperms, and angiosperms [10–12]. Within this large, diverse lineage, known as the lignophytes, numerous alternatives to this regular growth have evolved [13–16]. Modifications from the regular growth may derive from a single cambium with differential activity across its girth and/or multiple cambia [15, 17–19]. These alternative patterns of secondary growth produce diverse and complex stems architectures, also known as cambial variants [17, 18]. Many types of cambial variants are found in lineages containing lianas, although they also occur in trees, shrubs and herbs, aerial and underground organs [19, 20]. While the development of certain cambial variants has been investigated in recent years, the evolutionary history underlying the formation of these complex patterns is still being accumulated. The few previous studies integrating stem anatomical data with a phylogenetic framework have reviewed how disparate macromorphologies evolved from regular anatomies [8, 9], showing that their evolution involved different developmental mechanisms, especially heterochrony. Nevertheless, much has yet to be investigated to understand the total realm of changes in developmental trajectories that can contribute to the major complexity and diversity of the vascular system of plants in phylogenetically distant lineages.

Nyctaginaceae comprises c. 400 species that grows in a wide range of habits from arid deserts to tropical rain forests across the Americas, Africa and Indo-Pacific [21, 22]. The family includes herbs, shrubs, climbing plants, trees and suffrutescent species [21, 22]. Regardless of growth forms, the stem vascular anatomy of most Nyctaginaceae is remarkable for their polycyclic eustele [23] and cambial variants that appear in mature stems [24–26]. Most other plant families only have cambial variants in certain clades or their lianescent taxa (Malpighiaceae [27]; Sapindaceae [9, 28]), although they are also found in self-supporting plants derived from lianescent ancestors (Bignoniaceae [8]; Convolvulaceae [29]). According to Gianoli [30, 31], the evolution of climbing habit increased the species richness of clades compared to their non-climbing sister groups. However, since many clades containing climbing plants (mostly lianas) are also characterized for showing cambial variants, we asked whether the shifts in habit or the evolution of cambial variants is what explains the increase in species diversity. Given that Nyctaginaceae have a wide diversity of habits and because all lineages have cambial variants, the family is perfect to test if the transition to the lianescent habit or the evolution of cambial variants promotes species diversification or not.

Distinct types of cambial variants (i.e., interxylary phloem and successive cambia) have been reported for Nyctaginaceae and more recently, through detailed ontogenetic studies, taxa initially described as having successive cambia were demonstrated to have interxylary phloem instead [32]. However, for a long time, the understanding of anatomical and developmental diversity of the vascular system in Nyctaginaceae had been limited given the absence of ontogenetic studies in a broader taxonomic scale. Most previous work have focused on the ornamental charismatic taxa, such as *Bougainvillea* and *Mirabilis* [25, 26, 33, 34]. In this study, we investigated the developmental processes that generate disparate vascular architectures throughout the family which is likely independent of habits and habitats.

Here, we compared stem development in the context of a well-supported phylogenetic hypothesis to understand how developmental processes evolved over time and shaped the diversity of stem architectures in Nyctaginaceae. Among the findings we highlight: (i) the anatomical changes underlying the evolution of four ontogenetic trajectories in stem development; (ii) the anatomical, developmental and evolutionary lability of vascular meristems, especially the vascular cambium; and (iii) the significance of developmental mechanisms for evolutionary diversity of stem anatomical architectures. In addition, we evaluated whether species diversification rates have changed in Nyctaginaceae to explore the potential impact of both the multiple transitions in vascular anatomies and the lianescent habit in the diversification of the family.

## Materials and methods

### Taxon sampling and anatomical analysis

This study represents the broadest taxonomic sampling for stem anatomical studies in Nyctaginaceae to date. Stem samples of 55 species (~ 75 specimens) from 25 genera were collected, representing all major clades within the family, based on the most recent phylogenies for the group [21, 22, 35, 36]. Specimens were obtained mostly from field collections in different countries in both North and South America (Additional file 1: Table S1). Additional samples were obtained from dried stems from either herbarium vouchers or wood collections (Additional file 1: Table S1).

Samples from living plants were harvested at different heights of the stem to ensure that different developmental stages would not be missed. For herbs, complete stems were collected; for shrubs, lianas and scandent-shrubs, samples were obtained at the base of the plant and at least three different heights towards the shoot apex. For trees, we collected trunk samples at c. 1.30 m height and at different heights of selected branches. See Additional file 1: Table S1 for information on stem diameter for each specimen.

For the ontogenetic analyses, 27 species belonging to 22 genera of Nyctaginaceae were selected to account for all the variation both in terms of their phylogenetic distribution and anatomical patterns (Additional file 1: Table S1). For these species, sections were taken from different internodes beginning at the shoot apex until reaching the fully developed stem. For the remaining species, analyses of adult stems (the most developed stem available, from fully grown plants) were carried out to ensure the cambial variant types studied previously in detail were consistent.

During field work the samples were immediately fixed in FAA 50 (10% formalin, 5% acetic acid, 50% ethanol) and then transferred to a solution of 70% ethanol [37]. Anatomical sections were obtained following two different procedures: (i) young and small stems samples were dehydrated in an ethanol series, embedded in Historesin (Leica Mycrosystem, Wetzlar, Germany), sectioned in a rotary microtome (Leica RM2145, Nussloch, Eisfeld, Germany), and stained in 0.05% toluidine blue in glacial acetic buffer at pH 4.7 [38]; (ii) adult and large samples were softened in 5% ethylenediamine for up to 2 days [39], embedded in polyethylene glycol 1500, sectioned in a sliding microtome (Leica SM2010R, Nussloch, Eisfeld, Germany) [40, 41] and double stained in 1% astra blue and 1% safranine [42]. Sections were mounted with coverslip in Canada Balsam or Entellan® synthetic resin (Merck KGaA, Darmstadt, Germany) to make permanent slides.

### Phylogenetic framework, ancestral state reconstructions

To estimate the evolutionary history of ontogenetic pathways, we applied an ancestral state reconstruction using the same phylogenetic tree applied by Cunha Neto et al. [23] under Maximum Likelihood (ML) assumptions as implemented in Mesquite version 3.5 [43].

### Diversification analysis

#### Divergence times

To estimate the age of Nyctaginaceae, we conducted a Bayesian inference with BEAST v.2.6.5 [44], using two secondary calibrations derived from a thorough study of the divergence times of the angiosperm families [45]. We applied a uniform prior distribution to calibrate the root of the tree corresponding to the stem age of a group comprising Gisekiaceae and Nyctaginaceae, where the maximum value of the distribution was 83.6 Ma (Million years ago), and the minimum value was 52 Ma. We also applied a uniform prior distribution to calibrate the crown node of Nyctaginaceae, with a maximum value of 47.59 Ma and minimum value of 18.12. In BEAUti, we assigned a molecular substitution model as GTR + G, using empirical base frequencies, molecular clock set as uncorrelated with rates obtained from a log-normal distribution (UCLN; [46]), and a birth–death tree prior. We ran two independent analyses, each with 400 million generations, sampling parameters every 10,000 generations. We corroborated the correct mixing of the Markovian chains in Tracer v.1.6 [47], where the Effective Sample Size (ESS) was equal or higher than 200 for all the parameters. We obtained the Maximum Clade Credibility (MCC) tree with TreeAnnotator v.2.6.5 (beast2.org/treeannotator). The analyses in BEAST2 were performed in the server BEAGLE of the Instituto de Biología (Universidad Nacional Autónoma de México). The monotypic *Caribea littoralis* Alain was excluded from the diversification analysis given the poor morphological and phylogenetic information on this enigmatic taxon [35].

#### Diversification rate estimation

Using the time-calibrated phylogeny (MCC tree), we evaluated whether there have been changes or shifts in the diversification rate through time and among lineages. The diversification rate corresponds to the net number of species/lineages generated per time unit (speciation) considering the extinction [48, 49]. For this, we implemented a Bayesian analysis of macroevolutionary mixtures (BAMM v.2.5.0; [50]). BAMM estimates diversification rate shifts under a compound Poisson process through time and among lineages, using reversible-jump Markov chain Monte Carlo (rjMCMC) samplers to evaluate models that vary in the number of shifts proposed [50]. We selected a set of priors calculated in the R package BAMMtools [51, 52] for the speciation and extinction initial values. We specified a proportion of taxon sampling to consider the missing species of Nyctaginaceae and outgroups. We ran the analysis for 100 million generations. We evaluated the convergence of chains and with the package coda [53] we corroborated that the ESS of the MCMC was 200 or above.

### Trait-dependent diversification

To directly evaluate the contribution of characters in the diversification of Nyctaginaceae, we applied the Hidden State Speciation and Extinction method (HiSSE v.2.1.1; [53, 54]) for three characters, habit (self-supporting or climbing), eustele type (regular or polycyclic), and secondary growth (regular or variant). Using the dated phylogeny, we tested five models that varied in the relationship of the diversification rate and the observed, focal character. First, we evaluated a model, where diversification rates do not change across the phylogeny (Null). The second model tests two regimes of diversification rate that directly depend on the focal trait (BiSSE-like model), similar to the BiSSE method [55]. The third model evaluates the relationship of the focal trait and another unobserved character (HiSSE, with two hidden states). This is to evaluate the relative contribution of the focal trait to the diversification. Finally, following Beaulieu and O’Meara [54], we tested two models of character-independent diversification (CID) allowing diversification rate to vary across the phylogeny, but independently from the focal trait. One of these models has two parameters of diversification rate (CID2), and the other has four (CID4), comparable to BiSSE-like and HiSSE models, respectively. Net diversification rate was obtained through turnover rate (default) and the extinction fraction. In all models, the extinction fraction was constrained to have the same rate for all the character states, the free parameters were the turnover rate and the transitions between character states. Missing species were considered in the models by accounting for the proportion of sampled species relative to the existing species displaying each of the two states. To select models that better explain the variation of the data, we obtained the Akaike weights to compare the relative likelihood of each model. Character data set (Additional file 2: Table S2) and parameter settings (Additional file 3: R script) can be found in Additional files 2, 3.

### Terminology

As the range of terms related to the vascular system in Nyctaginaceae is highly diverse, we here define the terminology used in the present study (see Glossary).

## Results

### Four ontogenetic pathways link procambium to cambium and cambial variants

**Fig. 1:**
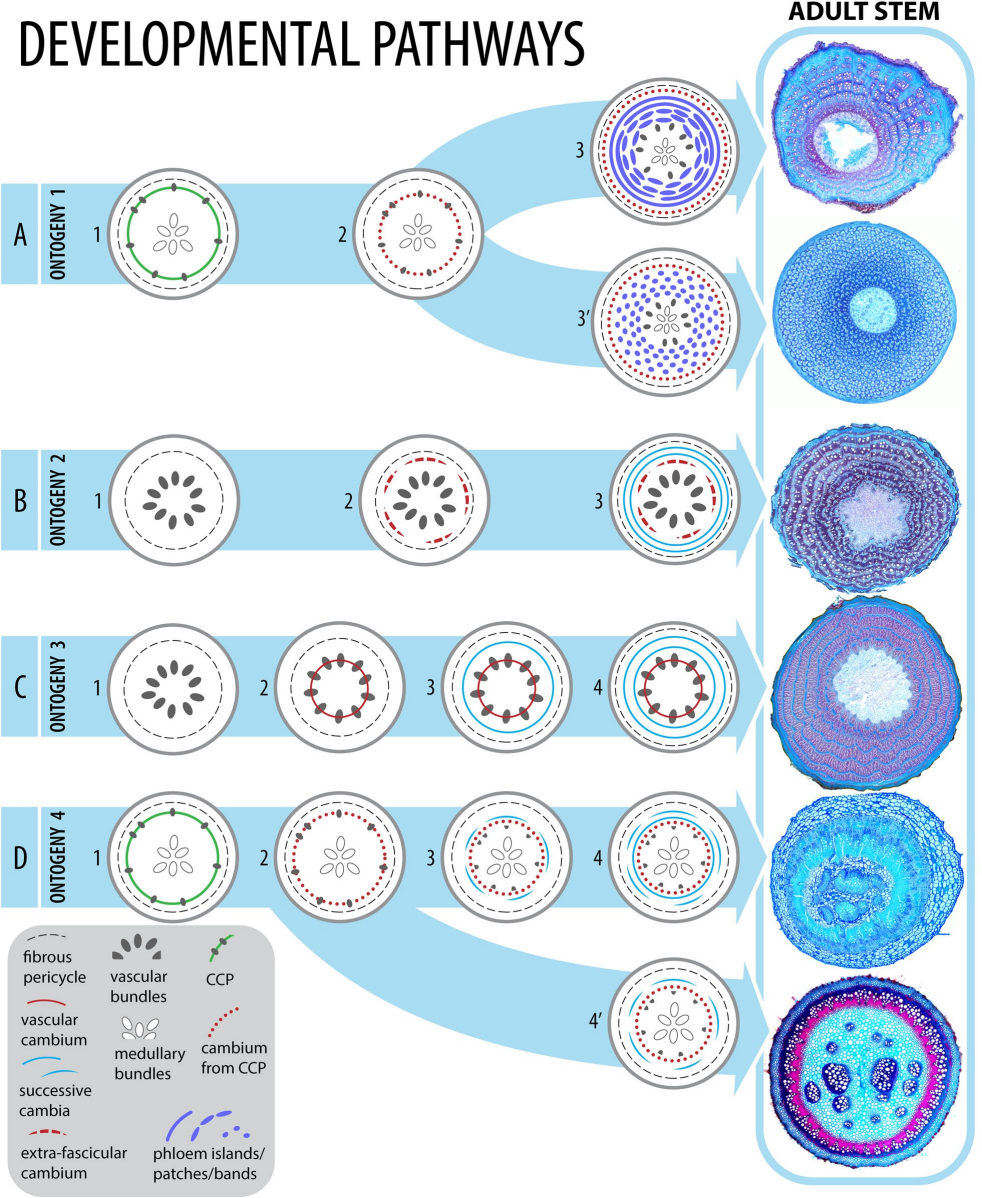
Diversity of stem ontogenies in Nyctaginaceae, illustrating developmental steps from eustele types to cambial variants. **A** Ontogeny 1 (interxylary phloem): polycyclic eustele, cambium from the CCP, phloem strands with different arrangements, illustrated by *Colignonia glomerata* (upper) forming bands and *Pisonia aculeata* (lower) forming phloem islands. **B** Ontogeny 2 (successive cambia): regular eustele, extra-fascicular cambium derived from the pericycle, additional successive bands or rings; *Leucaster caniflorus.* **C** Ontogeny 3 (successive cambia): regular eustele, regular cambium, new cambium formed de novo from the pericycle, additional successive bands or rings; *Reichenbachia hirsuta.* **D** Ontogeny 4 (successive cambia): polycyclic eustele, regular cambium derived from the CCP, new cambium formed de novo from the pericycle, additional small bands or rings of successive cambia; *Allionia incarnata* (upper) and *Okenia hypogaea* (lower). Drawing: Marcelo Kubo

In Nyctaginaceae, the stems may present two types of eustele, the regular or the polycyclic (with medullary bundles) (Fig. 1). The vascular system is also characterized by a distinguishable pericycle that can be uni- to multiseriate and which is divided into a portion of lignified cells and other that remains parenchymatous (Fig. 1). In mature stems, two types of cambial variants can be recognized, i.e., successive cambia and interxylary phloem, which derive from four different ontogenies (Fig. 1). Interestingly, representatives of all lineages of the family present variant vascular anatomies during secondary growth. Below we detail each of these ontogenies.

### *Ontogeny 1* (summarized from Cunha Neto et al. [32])—Steps: (i) polycyclic eustele, (ii) vascular cambium, (iii) phloem strands, and (iv) interxylary phloem (Figs. 1A, 2A–E)

**Fig. 2:**
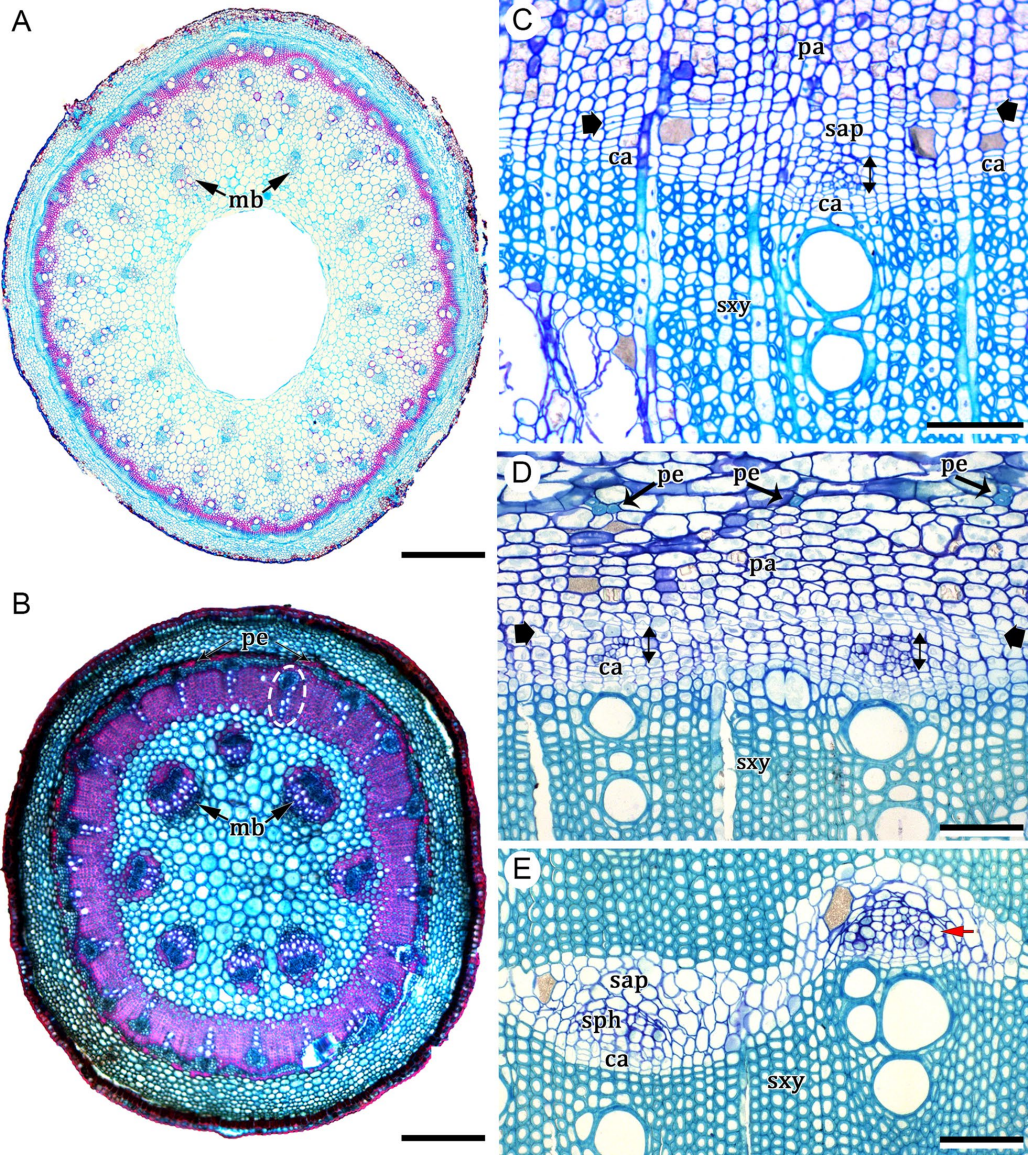
Development of interxylary phloem (ontogeny 1). **A** *Colignonia glomerata*, young stem showing polycyclic eustele and the transition from primary to secondary growth. **B** *Guapira pernambucensis*, stem in early secondary growth; note the vascular bundles (ellipse) formed by the CCP, whose phloem will be the first phloem island. **C** *Pisonia aculeata*, irregular activity of the cambium which results in phloem islands (double arrows) after the development of the coalescent (arching) cambium (thick arrows). Note that the coalescent cambium is originated from the axial phloem parenchyma in continuity with the original cambium. **D**, **E** *Guapira pernambucensis.* **D** Arching cambium enclosing two phloem islands. **E** Two phloem islands formed by secondary conducting phloem and sheathing axial parenchyma. The red arrow indicates a sieve-tube element. Scale bars: 200 μm (**A**–**C**); 100 μm (**D**, **E**). *ca* cambium, *mb* medullary bundles, *pa* axial phloem parenchyma, *pe* pericyclic fibers, *sap* sheathing axial parenchyma, *sph* secondary conducting phloem, *sxy* secondary xylem. **A**, **B** Stained with astra blue and safranin. **C**, **E** Stained with toluidine blue

This developmental pathway begins with a polycyclic eustele (reviewed by Cunha Neto [23])—medullary bundles + continuous cylindrical procambium (CCP) (Figs. 1A and 2A). This CCP is constituted by fascicular regions that produce vascular bundles, and interfascicular regions; both produce the cambium (Fig. 2A, B). After vascular bundles are formed from the CCP, a cambium is established from the procambium between primary xylem and phloem (Fig. 2B). This cambium presents an irregular activity leading to the formation of secondary xylem and phloem derivatives at different rates along the stem circumference, which results in the formation of phloem strands (Fig. 2C). Subsequently, these phloem strands are overarched by cambial segments originated by differentiation of the axial phloem parenchyma, called the coalescent (arching) cambium, and formed in continuity with the main cambium (Fig. 2C). This cambium produces secondary xylem inwards and phloem outwards. These new tissues enclose the islands of phloem, constituted mainly by conducting cells and axial phloem parenchyma (Fig. 2C, D) which here we denominate sheathing axial parenchyma (Fig. 2E). This process occurs repeatedly in the cambial zone and, as a result, many phloem strands are formed with the original cambial segment embedded within the secondary xylem (Fig. 2E).

The cambial variant described above characterizes the formation of interxylary phloem. In Nyctaginaceae, this pattern produces disparate stem architectures ranging from well-defined phloem islands with less sheathing axial parenchyma to long concentric bands of phloem and much sheathing axial parenchyma arranged tangentially (Fig. 1A). Intermediates between these two types forming phloem strands produced by longer coalescent cambium and confluences of phloem strands (patches) also occur.

Ontogeny 1 is the most common type within Nyctaginaceae, occurring in genera and species of various growth habits and from five out of the seven tribes, i.e., Boldoeae, Bougainvillea, Colignonieae, Nyctagineae and Pisonieae.

### *Ontogeny 2*—Steps: (i) regular eustele, (ii) extra-fascicular cambium, and (iii) successive cambia (Figs. 1B, 3A–E, 4A–C)

**Fig. 3:**
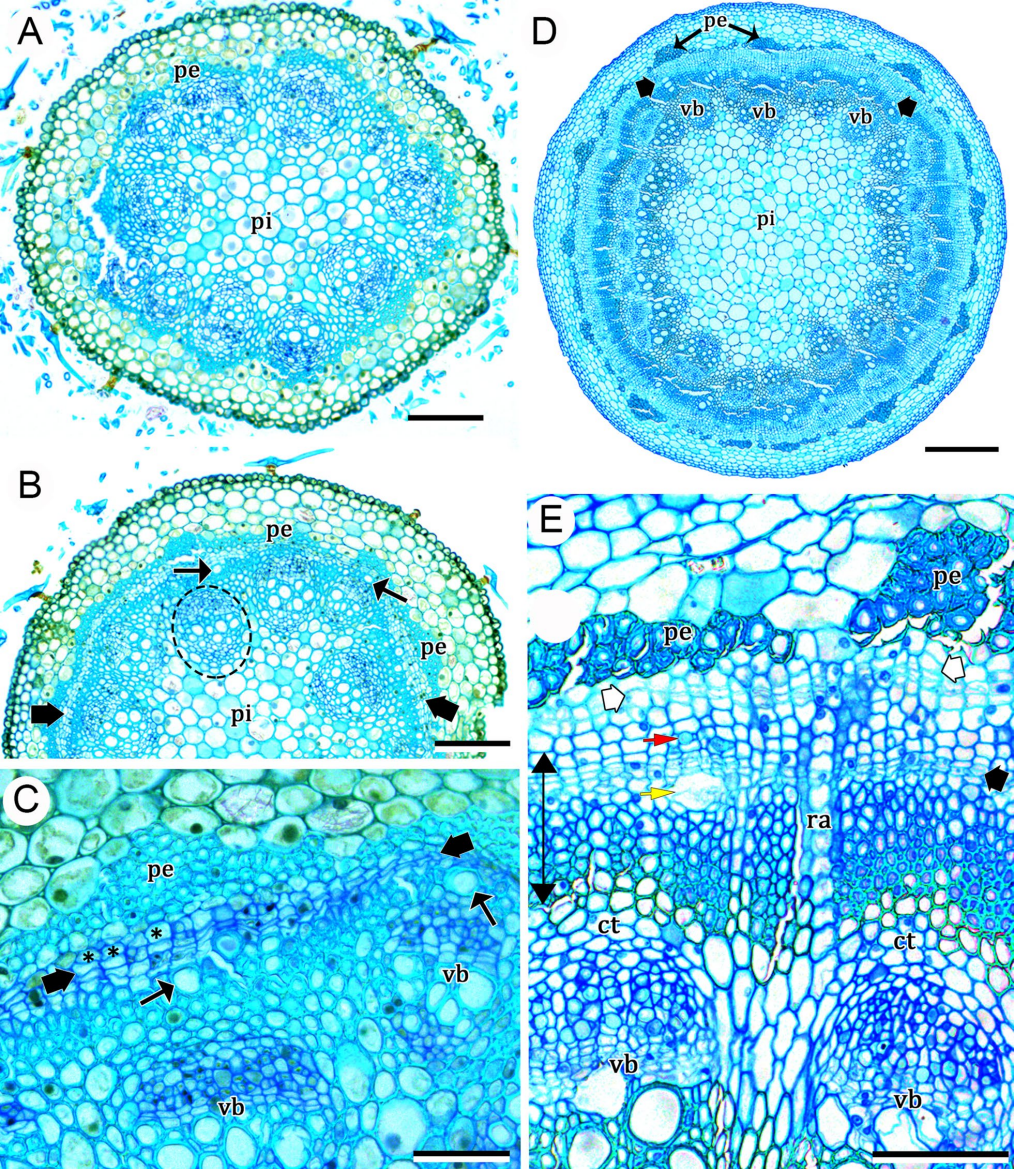
Development of successive cambia in stems of *Leucaster caniflorus* and *Ramisia brasiliensis* (ontogeny 2)*.* **A**–**C** *Leucaster caniflorus.* **A** Young stem with regular eustele. **B** Initiation of secondary growth through the extra-fascicular cambium (thick black arrows); note the first formed vessels of the secondary xylem (thin black arrows). **C** Detail of the extra-fascicular cambium (thick black arrows) and first formed vessels (thin black arrows); note the pericycle parenchyma cells (asterisks). **D**, **E** *Ramisia brasiliensis.* **D** Establishment of the extra-fascicular cambium (thick black arrows) outside the vascular bundles of the eustele. **E** Secondary vascular tissue (double arrow) originated from the extra-fascicular cambium (thick black arrow). The yellow arrow indicates a vessel element, and the red arrow indicates a sieve-tube element and its companion cell. Note the developing meristematic zone (white arrows) undergoing cell divisions that will give rise to the subsequent ring of successive cambia. The conjunctive parenchyma is also observed. Scale bars: 100 μm (**A**, **B**, **E**); 50 μm (**C**); 400 μm (**D**). *ct* conjunctive parenchyma, *pe* pericyclic fibres, *pi* pith, *ra* ray, *vb* vascular bundles of the regular eustele. **A**–**E** Stained with toluidine blue

**Fig. 4:**
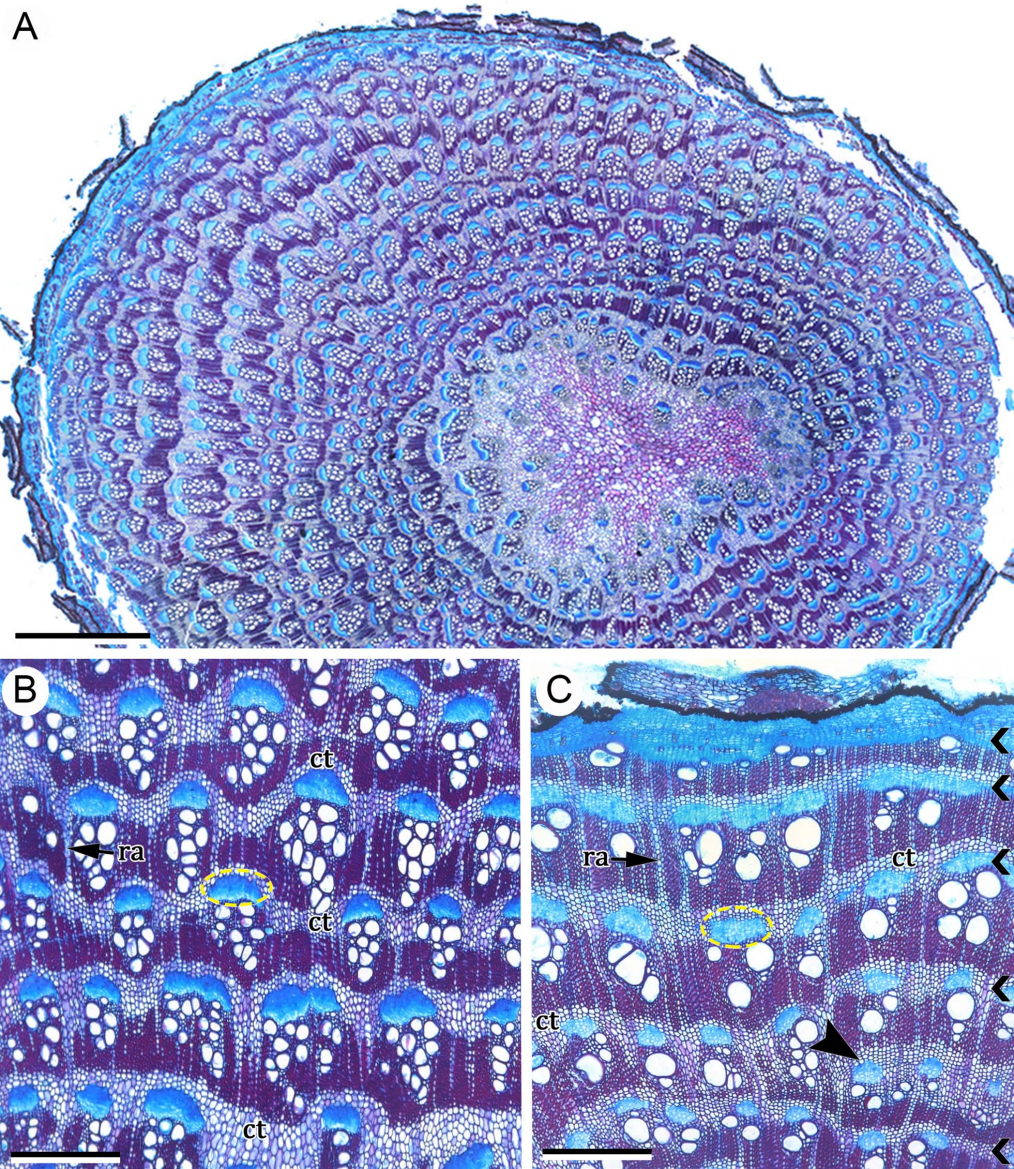
Cross view of developed stems with successive cambia (ontogeny 2). **A**, **B** *Andradea floribunda*. **C** *Leucaster caniflorus.* **A** Adult stem showing several rings of successive cambia. **B** Detail of the successive cambia and their vascular products; the phloem form small strands (yellow ellipse). Note the wavy appearance of the vascular tissues. **C** Rings of successive cambia in more or less regular concentric arrangement (pointers); the arrowhead indicates an incomplete segment between the other upper and lower rings; note the phloem forming small strands (yellow ellipse). Scale bars: 2000 μm (**A**); 500 μm (**A**, **B**). *ct* conjunctive tissue, *pe* pericyclic fibres, *pi* pith, *ra* ray. **A**–**C** Stained with astra blue and safranin

This pattern differs from ontogeny 3 (see description below) for not forming a regular cambium (derived from the procambium remnants of vascular bundles), even though it starts stem development with a regular eustele (Fig. 3A). The genera under this ontogeny lack medullary bundles. Instead of forming a regular cambium, the first cambium differentiated externally to the vascular bundles (i.e., extra-fascicular cambium), giving rise to secondary xylem produced internally and secondary phloem formed to the outside (Fig. 3B–E). This variant cambium differentiates from a meristematic zone formed by divisions of the pericyclic parenchyma cells located between the primary phloem and the fibrous pericycle (Fig. 3C). Subsequently, additional cambia are formed outwards from remaining cells of the previous meristematic zone (Fig. 3E), whereas some parenchyma cells produced by the meristematic zone will constitute what here we call tangential conjunctive tissue between two vascular increments (Figs. 3A, 4A–C). At maturity, the increments of successive cambia can appear wavy (Fig. 4B), or more or less concentric (Figs. 1C, 4C). In any case, sieve-tube elements and their companion cells are formed always at the opposite side of the vessel elements, while conjunctive parenchyma form an intricate network with the vascular rays (Fig. 4B–C).

Ontogeny 2 was observed in the genera *Andradea, Leucaster* and *Ramisia,* which are trees or lianas and all belonging to tribe Leucastereae.

### *Ontogeny 3*—Steps: (i) regular eustele, (ii) vascular cambium, (iii) installation of variant cambium, and (iv) successive cambia (Figs. 1C, 5A–D, 6A–D)

**Fig. 5:**
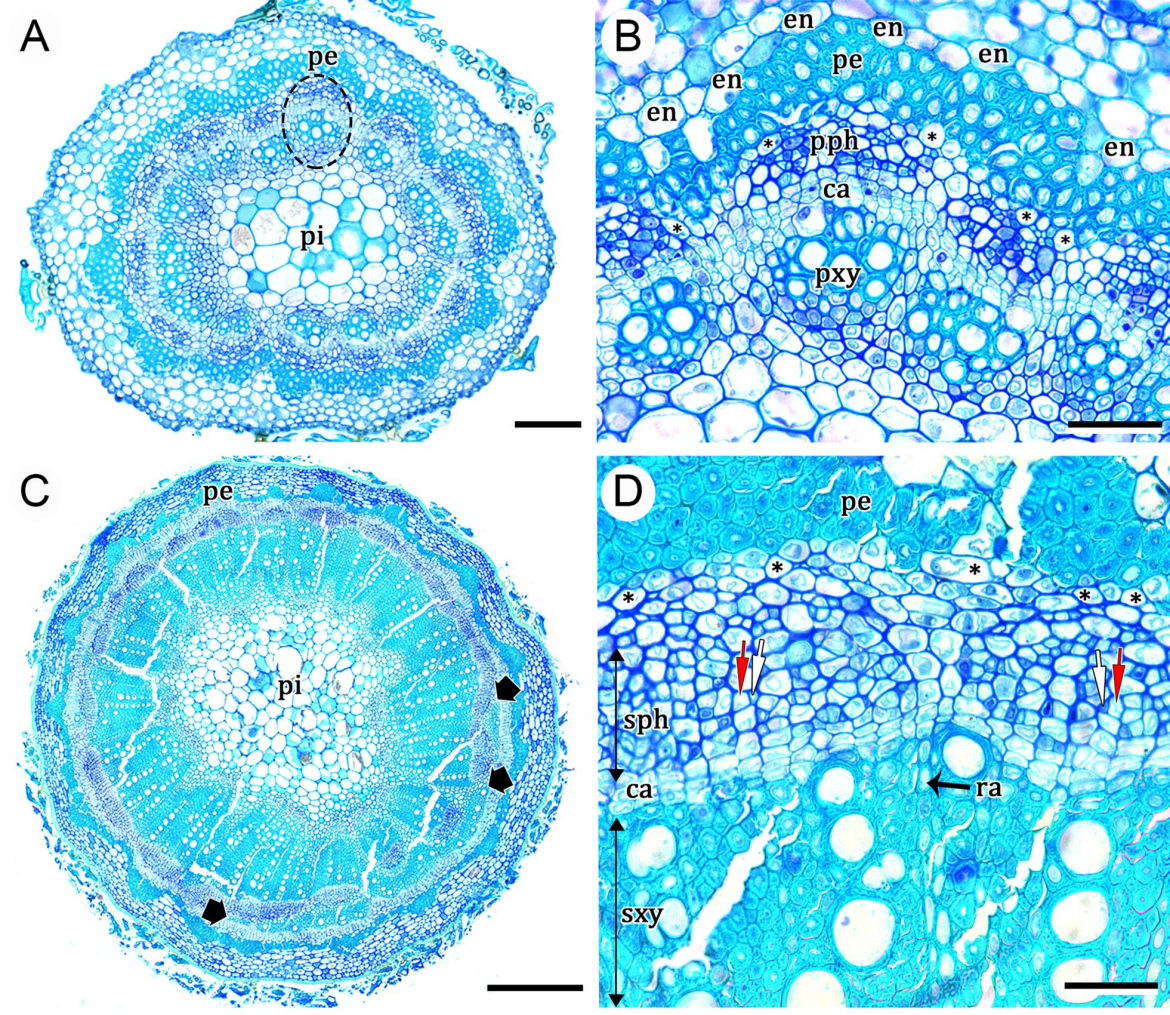
Early stem development in *Reichenbachia hirsuta* (ontogeny 3)*.* **A** Initial secondary growth derived from a regular eustele; the dashed ellipse indicates the position of a vascular bundle. **B** Detail of regular cambium and its derivatives, primary vascular tissues, fibrous pericycle and parenchymatous pericyclic cells (asterisks). **C** Stem during regular secondary growth, first variant cambium and its derivatives (thick arrow); see details in next Fig. 6A–D. **D** Regular cambium, secondary xylem and secondary phloem. Scale bars: 50 μm (**A**–**C**); 400 μm (**D**). Arrow (white), companion cell; Arrow (red), sieve-tube element; Asterisks, pericyclic parenchyma cells; *ca* regular cambium, *en* endodermis, *mz* meristematic zone, *pe* pericyclic fibres, *pi* pith, *pph* primary phloem, *pxy* primary xylem, *ra* ray, *sph* secondary phloem, *sxy* secondary xylem. **A**–**D** Stained with toluidine blue

**Fig. 6:**
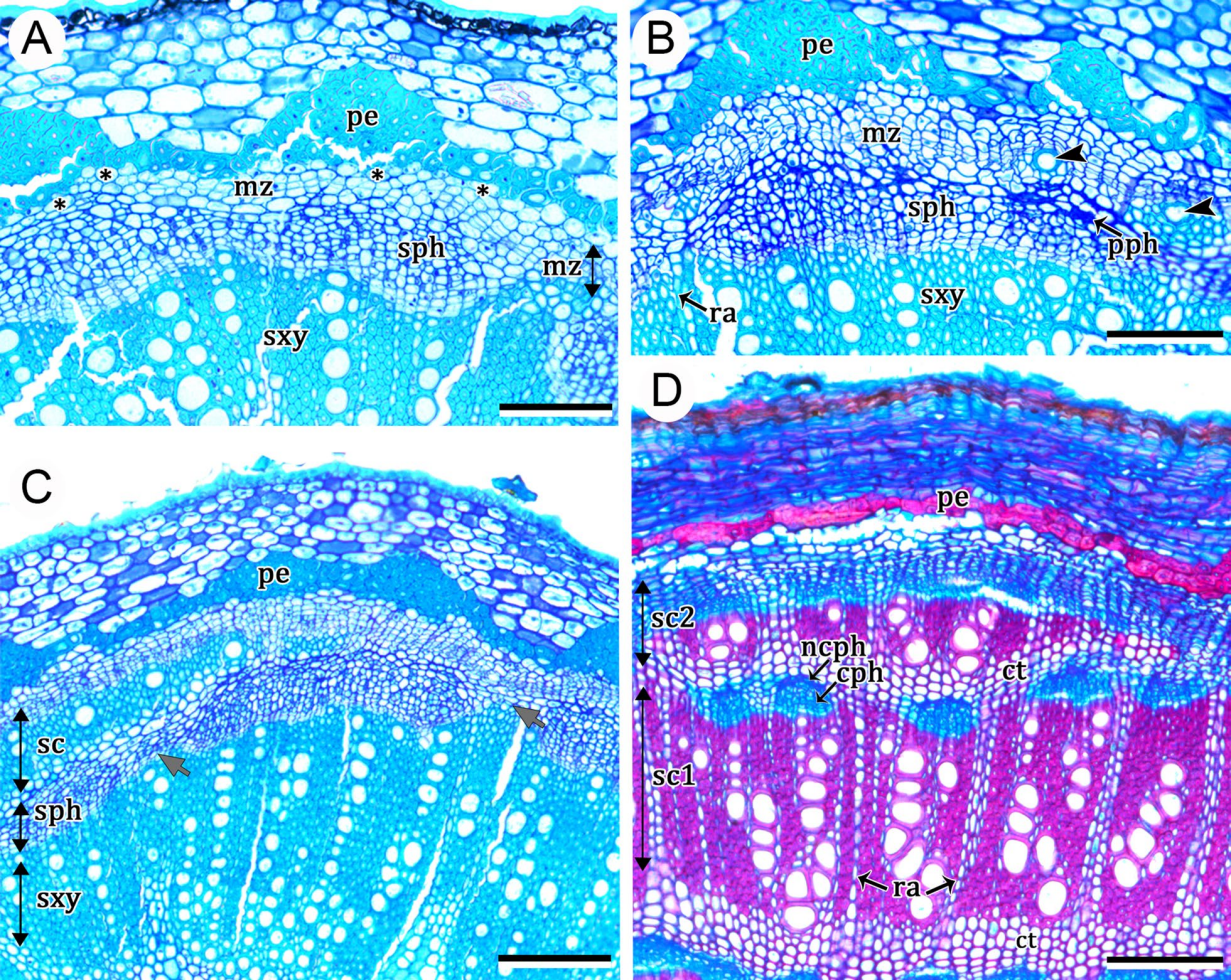
Development of successive cambia in stems of *Reichenbachia hirsuta* (ontogeny 3). **A** Meristematic zone and pericyclic cells that remain parenchymatous (asterisks). **B** Development of the first variant cambium within the meristematic zone. Arrowheads indicate vessel elements. **C** First ring of successive cambia with their variant xylem and variant phloem (sc). Initially, the interfascicular cambium (grey arrow) produces mostly phloem axial parenchyma to the outside and xylem fibers to the inner side. **D** Mature stem showing two rings of successive cambia with conjunctive tissue between them. Scale bars: 100 μm (**A**–**C**); 200 μm (**D**). *cph* conducting phloem, *ct* conjunctive tissue, *ncph* non-conducting phloem, *mz* meristematic zone, *pe* pericyclic fibres, *pph* primary phloem (crushed), *ra* ray, *sc* sc1 e sc2, increments of successive cambia, *sph* secondary phloem, *sxy* secondary xylem. **A**–**C** Stained with toluidine blue. **D** Stained with astra blue and safranin

This ontogeny initiates with a regular eustele (Fig. 5A). Similarly to species in ontogeny 2, the species under this ontogeny lack medullary bundles. Later, a regular cambium develops from the fascicular and interfascicular cambium and starts to produce secondary tissues in the usual way, i.e., secondary xylem centripetally and secondary phloem centrifugally (Figs. 5A–D, 6A–D). Initially, the interfascicular cambium may produce mostly phloem axial parenchyma to the outside and produce xylem fibres, vessel and rays to the inside (Fig. 6C). After some period of regular growth, a new meristematic zone arises through subsequent divisions of pericyclic parenchyma cells outside of the primary phloem (Fig. 6A–C). Then, a new cambium (variant cambium) differentiates in the middle of the meristematic zone (Fig. 6B), producing secondary xylem and secondary phloem in the usual polarity (Fig. 6C, D). Subsequently, by the same mechanism of the first variant cambium, new cambia arise successively in centrifugal, concentric order, each originating from the outer derivatives of the preceding meristematic zone (Fig. 6D).

In mature stems, the tangential conjunctive tissue forms a network with narrow and wide vascular rays (Fig. 6E). The sieve-tube elements and their companion cells form mostly strands surrounded by the tangential conjunctive tissue, located at an opposite pole of the radially arranged vessel elements (Fig. 6E, F).

Ontogeny 3 was observed exclusively in the shrubby species of genus *Reichenbachia* (tribe Leucastereae).

### *Ontogeny 4*—Steps: (i) polycyclic eustele, (ii) vascular cambium, (iii) installation of variant cambium, and (iv) successive cambia (Figs. 1D, 7A–E)

**Fig. 7:**
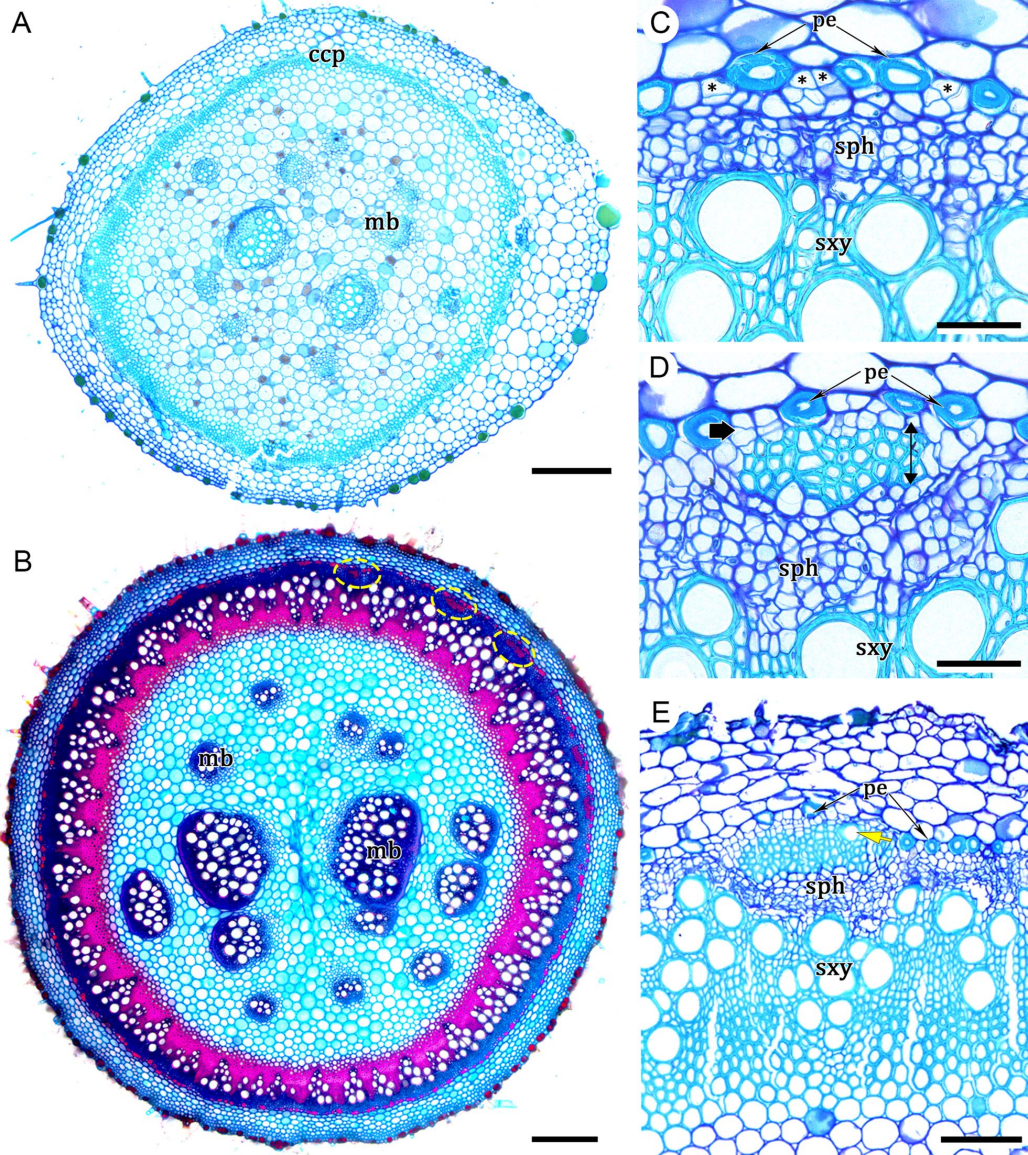
Development of successive cambia in stems of *Okenia hypogaea* (ontogeny 4)*.* **A** Primary growth with polycyclic eustele. **B** Stem with cambial variant. Dashed ellipses indicate the arcs of successive cambia; note medullary bundles in the pith. **C** Secondary xylem and secondary phloem, pericycle parenchyma undergoing cell division (asterisks) to form a meristematic zone, where new cambia will be formed. **D** Developing variant cambium (thick arrow) and variant xylem formed mostly by fibres (double arrow). **E** Developing arc of successive cambia showing variant xylem with first formed vessel (yellow arrow). Scale bars: 200 μm (**A**); 400 μm (**B**); 50 μm (**C**, **D**); 100 μm (**E**). *ccp* cylindrical continuous procambium, *mb* medullary bundles, *pe* pericyclic fibers, *sph* secondary phloem, *sxy* secondary xylem. **A**, **C**–**E** Stained with toluidine blue. **B** Stained with astra blue and safranin

This ontogeny differs from ontogeny 3 for having a polycyclic eustele (Fig. 7A). As for ontogeny 1 (also with polycyclic eustele), a vascular cambium develops from the fascicular and interfascicular CCP, but in this case the cambium produces regular secondary xylem and phloem at relatively regular rates for some time (Fig. 7B, E). Later, divisions of the pericyclic parenchyma cells (Fig. 7C) form a meristematic zone, where new segments of cambia develop producing new vascular increments composed of variant secondary xylem and phloem in the usual polarity (Fig. 7D, E).

Ontogeny 4 was found only in two monotypic genera (*Allionia* and *Okenia*) of herbaceous plants from tribe Nyctagineae*.* Although successive cambia establish in these plants, these species show relatively little secondary tissues even in the most developed stems, as seen in *Allionia incarnata* (Fig. 1D; reviewed by Cunha Neto [56]) and *Okenia hypogaea* (Figs. 1D, 7B). In *Allionia,* one or two complete rings of vascular increments are possible, whereas in *Okenia hypogaea,* only small cambial segments of first order were seen in the most developed stems (Figs. 1D, 7B).

### Character mapping and ancestral state reconstruction

**Fig. 8:**
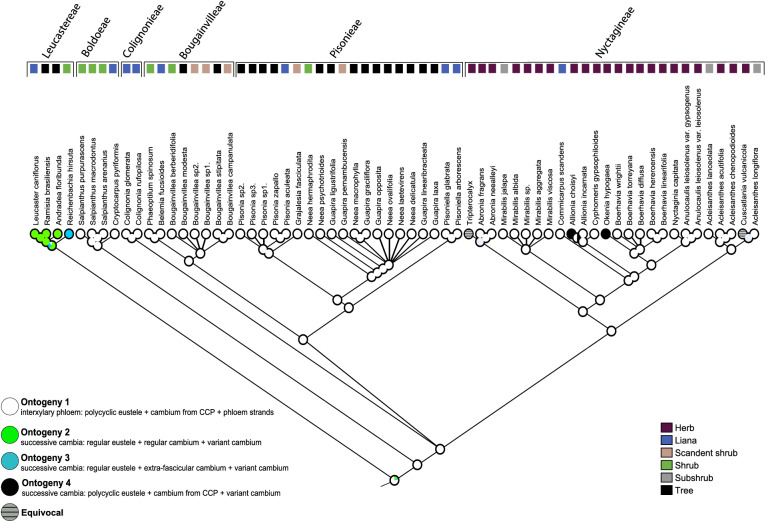
Maximum likelihood reconstruction of ontogenetic pathways mapped with Mesquite on a phylogenetic tree of Nyctaginaceae following the same topology used by Cunha Neto et al. [23]

To assess the evolution of stem development in Nyctaginaceae, the ontogenies were mapped onto the current phylogeny of the family. Each of the four ontogenetic pathways were delimited as character states. The phylogenetic analysis showed that ontogeny 1 (polycyclic eustele + interxylary phloem) is the most common and was reconstructed as the ancestral state (79% presence), with a few secondary losses (Fig. 8, e.g., Leucastereae, Nyctagineae). Ontogeny 4 (polycyclic eustele + successive cambia) evolved twice, being found in genera belonging to Nyctagineae that includes the majority of the herbaceous species, whereas ontogeny 2 (regular eustele + regular cambium + successive cambia) and ontogeny 3 (regular eustele + extra-fascicular cambium + successive cambia) evolved each only once, occurring in genera of tribe Leucastereae (the clade sister to the rest of the family) which is formed by shrubs, trees and/or lianas.

### Divergence times and diversification rate estimation

**Fig. 9:**
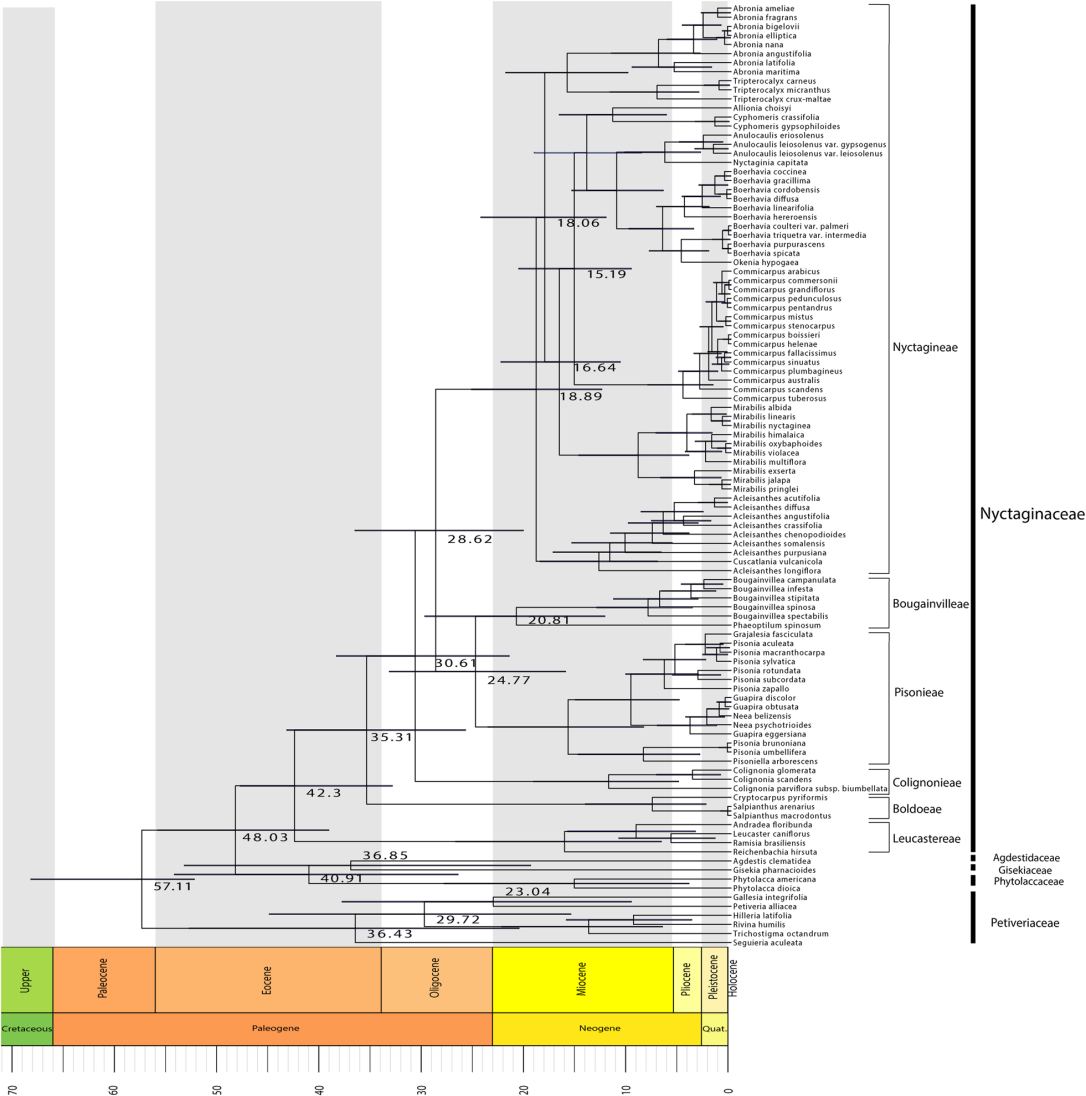
Maximum clade credibility tree (MCC) with divergence time estimates for Nyctaginaceae and related families. Numbers correspond to mean age estimates (in million years). Bars indicate age confidence intervals from a 95% highest posterior density (HPD)

We estimated the age of divergence of Nyctaginaceae and close relatives with BEAST2 (Fig. 9). We obtained the Maximum Clade Credibility tree (Fig. 9, Additional file 4: Nexus tree), which shows the mean age for the main nodes in the phylogeny. Table 1 shows the mean crown age estimates for the major clades and its associated credibility interval represented by the 95% Highest Posterior Density (HPD). Nyctaginaceae probably diverged from its sister group (stem age), Phytolaccaceae, 48 Ma (HPD: 38.97–55.56 Ma) and diversified (crown age) 42.3 Ma (HPD: 32.82–47.59 Ma), both events occurring at the Middle Eocene.

**Table 1:** Divergence-time estimation of mean crown ages in Million years (Ma)

Family	Crown age (Ma)	95% HPD
Nyctaginaceae	42.3	32.82–47.59
Phytolaccaceae	40.91	26.45–53.95

Highest posterior density intervall (95% HPD)

**Fig. 10:**
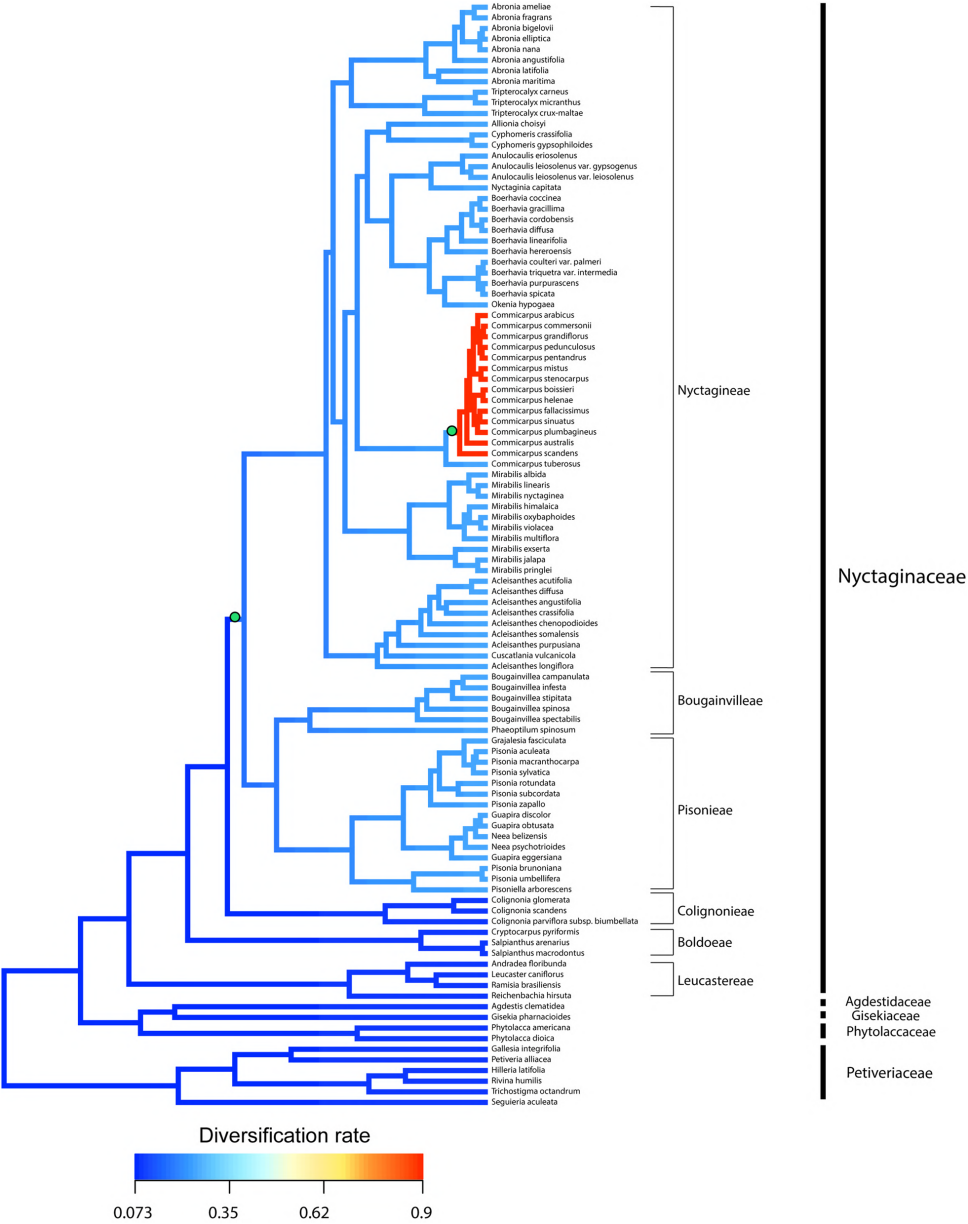
Net diversification rate dynamics in Nyctaginaceae and most-closely related families estimated by BAMM. Branch color reflects the mean of the marginal posterior density of net diversification rates for each segment of the branches, with rates increasing from blue to red. Two green circles indicate the most probable rate shift configuration found using BAMM

The estimation of diversification rate shifts implemented in BAMM resulted in a set of configurations, each configuration represents a group of possible rate shifts occurring in the phylogenetic tree at different times. The Maximum a posteriori probability (MAP) configuration was obtained in the R package BAMMtools and is observed in the phylorates plot shown in Fig. 10, where the estimated rate at each segment of the branches is the mean of the marginal posterior density of the diversification rate, and the shifts are marked by a green circle. The MAP includes the most frequent configuration with two shifts in the diversification rate (i.e., speciation minus extinction; green circles in Fig. 10; check Additional file 5: Fig. S1 for all most credible shift sets recovered by BAMM). The results indicate one diversification shift derived from a rise in the speciation rate alone in the clade that includes Pisonieae–Bougainvilleae–Nyctagineae, and another diversification increase derived from rises in both speciation and extinction rates in bulk of *Commicarpus*, as observed in the speciation rate and in the extinction rate separately.

**Fig. 11:**
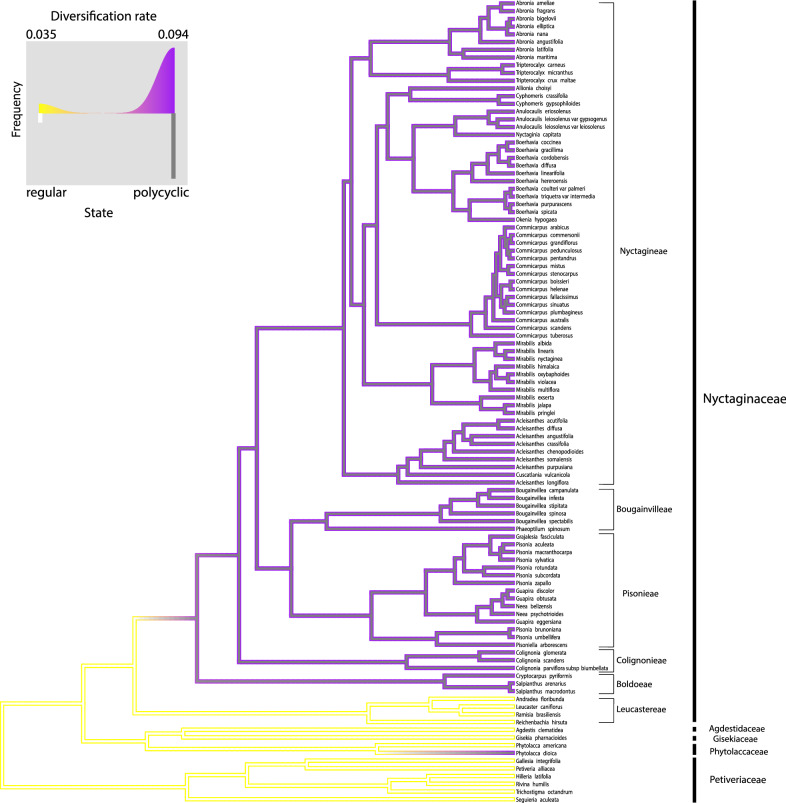
Diversification rate associated to eustele type. Diversification rate and character state transition correspond to the preferred model, BiSSE-like. Diversification rate is represented by branch contour color: yellow = lower rate, purple = higher rate. Character states are represented by branch filling color: withe = regular, gray = polycyclic. Legend shows the histograms of the frequency of species with one of the two states and their association with diversification rate

Table 2 shows the results from the trait-dependent diversification analysis and model comparison. For habit and secondary growth, the preferred model was the character-independent diversification (CID2). In turn, for eustele type, the preferred model was a trait-dependent diversification with two diversification regimes (BiSSE-like). This indicates that eustele type had a relationship with diversification rate, being the polycyclic eustele associated to elevated diversification rate compared to regular eustele (Fig. 11).

**Table 2:** Model comparison from the trait-dependent diversification analysis

Model	Habit			Eustele type			Secondary growth		
	loglik	AICc	AICw	loglik	AICc	AICw	loglik	AICc	AICw
Null	− 366.56	741.53	0.00	− 322.19	652.79	0.02	− 320.53	649.47	0.00
BiSSE-like	− 366.53	743.66	0.00	− **316.99**	**644.58**	**0.91**	− 318.61	647.73	0.00
HiSSE	− 350.74	723.85	0.41	− 315.52	653.41	0.01	− 309.64	641.71	0.04
CID2	− **355.41**	**723.68**	**0.44**	− 318.70	650.28	0.05	− **311.45**	**635.79**	**0.84**
CID4	− 354.13	725.77	0.16	− 317.77	653.05	0.01	− 311.06	639.69	0.12

Bold rows indicate the preferred model for each evaluated character

*loglik* log-likelihood, *AICc* corrected Akaike score, *AICw* Akaike weight

## Discussion

### The evolution of cambial variants in Nyctaginaceae represents an example of continuum morphology

Because evolution can be seen as the transformation of ontogenies, many authors have argued that plant morphology is better understood under more dynamic and process thinking than the typological view of classical morphologists (e.g., Wilhelm Troll, Donald Kaplan) [57, 58]. One of the alternative worldviews known as continuum morphology has been built through the works of several botanists, such as Agnes Arber [59] and Rolf Sattler [3, 60, 61]. This approach implies that the static view of plants having structures (such as organs or the different variants here) with clear cut boundaries seems no longer sufficient to explain plant morpho-anatomical diversity [57]. In contrast, the process and continuum morphology recognize plants as combinations of developmental processes or as dynamic continua, based on the acceptance of the partial homology concept [59, 61], which reiterates the blurry boundaries (mixed identities, fuzzy morphology) between what used to be considered as separate structural categories [62, 63]. This means that diversity is more likely to result from quantitative rather than from qualitative differences in development, which leads to the identification of several intermediate forms between two categories [61]. The continuum approach may be as well the better way to look at the evolution of successive cambia from the interxylary phloem in Nyctaginaceae.

Although successive cambia and interxylary phloem are recognized in the literature as two types of cambial variants, their occurrence in Nyctaginaceae indicates that in some cases there is a blurry boundary delimitation between these two patterns—which are represented in two levels*.* First, plants with ontogeny 1 that are characterized with interxylary phloem may present phloem strands immersed within the xylem with intermediate-like arrangements, i.e., phloem islands, patches or bands. These arrangements result from different extensions of the coalescent cambium which is formed in continuity with the single cambium and encloses the phloem strands and sheathing axial parenchyma with diverse spatial distributions [32]. Given that in plants with bands the stem initiates forming phloem islands followed by patches and then bands, the development of this ontogeny itself indicates the existence of a continuum between these different stem macromorphologies. Second, the ontogenies characterized with successive cambia shows that the independent cambia form usually long tangential bands of vascular tissues similar to the interxylary phloem forming bands of other species of the family. The diverse topologies observed in plants with successive cambia which has received numerous attempts of subcategorization is another evidence of these blurry categories (Additional file 6: Table S4).

The continuum worldview has been applied especially in organ identity [62–65] but a parallel seems possible to be established with other biological systems (e.g., growth forms [66], photosynthetic pathways [67]), which are not framed around the distinctiveness of shoot organs*.* Here, for the first time, the diversity of cambial variants is interpreted under the concept of continuum morphology, although within the same organ. These observations for Nyctaginaceae enrich our understanding of these complex vascular morphologies, as it illustrates how ontogenies changed across evolutionary time producing intermediate forms that at some point can be distinguished as discrete categories.

### The origins and developments determining the cambial variants in Nyctaginaceae

The recognition of interxylary phloem along with the occurrence of successive cambia is based primarily on their differences in development, i.e., single vs. multiple cambia, respectively [32], but these developmental pathways result in similar anatomies and can be considered to integrate into intermediate forms in Nyctaginaceae.

Here we identified that to the four ontogenies, all events of vascular development originated internally to the pericyclic fibres. These findings contradict the hypothesis that the cambial variants in Nyctaginaceae are formed from a meristem arising in the cortex [26, 33, 34, 68, 69]. Instead, all cambial variants originated from procambial-derived cells, corroborating previous findings [56, 70]. Therefore, successive cambia and interxylary phloem are evolutionarily and developmentally linked in Nyctaginaceae, because both the cambium giving rise to phloem strands within the secondary xylem (interxylary phloem) and the meristematic zone producing a de novo cambium (successive cambia) may be traced back to the procambium at some point in stem development.

Although the cambial variants in Nyctaginaceae have similar origins at the cell lineage level (i.e., procambium-derived cells), the eustele types and subsequent events in their development are diverse, leading to four distinct ontogenies. In a previous work we showed that the origin and development of interxylary phloem in Nyctaginaceae is similar to other groups, except for the fact that in this family they initiate with a polycyclic eustele [32]. Although ontogenies 2, 3 and 4 are characterized as successive cambia at maturity, they present different developmental stages. The developmental steps for *Reichenbachia* (ontogeny 3) are the same described in most families with this cambial variant, i.e., a new cambium is formed de novo (mostly but not always) from the pericycle in stems with regular eustele. This is the case of species both in the gymnosperms (e.g., *Gnetum*, *Cycas*—[68]) and several families of angiosperms (e.g., Menispermaceae [71]; Convolvulaceae [29]; Sapindaceae [72]). On the other hand, successive cambia, as described in ontogenies 3 and 4, differ from the taxa mentioned above, because they either do not produce a regular cambium, forming an extra-fascicular cambium (ontogeny 2), or because the stem begins with a polycyclic eustele, and the first cambium is derived from the CCP instead of a regular cambium (ontogeny 4). It is important to highlight that the appearance of the extra-fascicular cambium, which is independent from the primary vasculature, corroborates the potential of perivascular tissues (i.e., the pericycle) to produce new meristems. Finally, this study also corroborates the idea that there are multiple origins (e.g., primary phloem, secondary phloem, cortex [29, 72–75]) and developmental trajectories that can lead to successive cambia, unlike the hypothesis of a universal phenomenon across different plant groups [68, 69, 76].

### Nyctaginaceae stands out for having all extant lineages characterized by variant anatomies

The ancestral state reconstruction presented here demonstrates that the ancestor of Nyctaginaceae already had cambial variant, with interxylary phloem (ontogeny 1) reconstructed as the most likely character state for the ancestral node of the family. This observation is remarkable, because it indicates that Nyctaginaceae is one of the few examples, where the cambial variants are present in all members and is shown to be shared with other members of the phytolaccoid clade, being likely plesiomorphic for Nyctaginaceae. In most families with cambial variants they appear only in one group, mostly in clades containing lianas or descending from lianas (e.g., Bignoniaceae, Convolvulaceae) [8, 9, 28, 77]. Contrary to expectations, interxylary phloem (ontogeny 1) is the most common type of cambial variant in Nyctaginaceae, occurring in five out of seven tribes, including genera, such as *Bougainvillea*, *Boerhavia*, *Mirabilis* and *Pisonia*, which used to be classified as having successive cambia (reviewed by [32]). The ontogenies appeared only once, except for ontogeny 4 that evolved twice. The evolution of ontogenies 2 and 3 in members of Leucastereae is interesting, because the tribe has other morphological (e.g., type of trichomes, pollen and fruit—[42, 76, 77]) and vascular characters (e.g., type of stele, [23]) that are exclusive if compared to other lineages of the family. Curiously, from all stem ontogenies of Nyctaginaceae, *Reichenbachia* (ontogeny 3) is the only taxon following the commonly described development for successive cambia, i.e., regular eustele + regular cylinder + successive cambia [29, 71, 72].

Ontogeny 4 is the only type with more than one evolution with two transitions in tribe Nyctagineae. The evolution of successive cambia in *Allionia* and *Okenia* is noteworthy for the fact that they are both small herbs with limited secondary growth in the regular cylinder, but the successive cambia still develop in some way. However, this might not be surprising, since the presence of cambial variants in other herbs (annuals or perennials) across the Caryophyllales is commonly observed, suggesting that if given time to grow, most of them can form additional vascular tissue in the form of cambial variants [69, 78].

The development and evolution of distinct patterns of cambial variants in Nyctaginaceae is remarkable, because successive cambia was thought to be the only cambial variant in the family [26, 34, 69, 78]. As hypothesized earlier, similar interxylary phloem as observed here may also be present in other caryophyllalean families [32]. As with other traits (e.g., floral morphology [79–81]), the presence of cambial variants likely share developmental and genetic programmes (deep homology) triggering the recurrent evolution of this morphological feature in multiple Caryophyllales lineages.

### Evolution of development: how different ontogenies generate similar stem macromorphologies

Because primary and secondary vascular tissue may have intrinsic developmental relations, the investigation of the diversity and evolution of vascular anatomies in Nyctaginaceae needs to include the products of procambium, cambium and cambial variants to thoroughly comprehend the anatomical and developmental shifts in stem ontogeny. Here the integration between ontogeny and phylogeny showed that adult stems with distinct cambial variants evolved from two different eustele types. Therefore, the evolution of patterns of secondary growth in Nyctaginaceae is built upon distinct primary vascular morphologies.

**Fig. 12:**
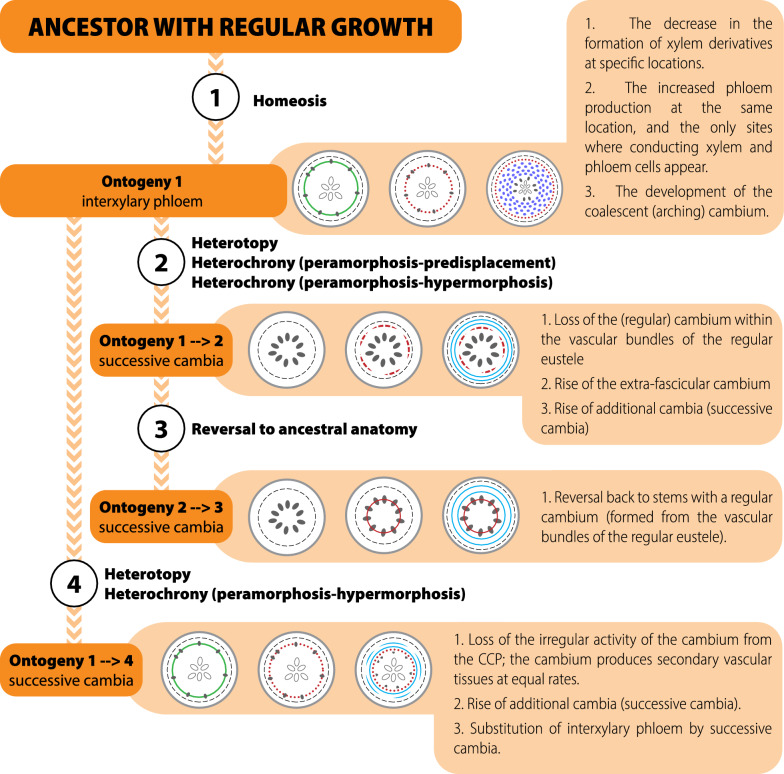
Overview of the anatomical modifications across evolutionary time in the stem vascular system of Nyctaginaceae and the evolutionary mechanisms generating their complex morphological diversity. Drawing: Marcelo Kubo

Evolutionary mechanisms are difficult to be interpreted for the evolution of vascular patterns in Nyctaginaceae because of multiple developmental transitions (Fig. 12). Here, three processes are inferred to generate the stem diversity found in the family (Fig. 12): homeosis, heterochrony, and heterotopy. (1) The formation of interxylary phloem (ontogeny 1) in relation to a putative ancestor with regular anatomy seems likely to represent a case of homeosis, since the unusual activity of the cambium leads to the presence of phloem strands in the place of secondary xylem. Similar cases of homeosis in woody plants has been hypothesized for example in species with parenchymatized xylem, that is, in cases, where non-lignified parenchyma occur where fibres, vessels and lignified axial parenchyma would be present (e.g., lianas, succulents) [7]. (2) In the evolution of ontogeny 2 from ontogeny 1, the extra-fascicular cambium appeared, suggesting a case of heterotopy, since the first vascular cambium arises in a different position from that present in the ancestor. In addition, at the structural level the development of ontogeny 2 is based on the earlier onset of formation of the cambial variant by suppressing one of the ontogenetic stages (i.e., formation of a cambium from the primary vascular system); therefore, it may also illustrate for the first time a case of predisplacement, a form of peramorphosis (heterochrony). In wood anatomy, most cases of heterochrony suggest the occurrence of prolonged juvenile characteristics into adult forms (paedomorphosis) [7, 82, 83], and a case of peramorphosis (hypermorphosis—evolution by developmental additions) is also suggested for the origin of successive cambia in *Paullinia,* Sapindaceae [9]. Moreover, because ontogeny 2 evolved from ontogeny 1, this transition also requires modifications in the primary vascular system which indicates developmental changes that are regulated by an independent developmental module [84]. Thus, modularity may also be a source for anatomical diversity in this group. The evolution of ontogeny 3 from ontogeny 2 implicates in the appearance of a regular cambium. This transition indicates that a partial regression to the state of the ancestor of the family occurred in this lineage, if we consider that the regular cambium occurs in the same position of the single cambium generating interxylary phloem. Similarly, the evolution of ontogeny 4 requires a reversion from the cambium with unusual activity to the regular cambium, and then a new cambium is formed constituting the successive cambia, which suggests an additional developmental event.

In the context of continuum morphology, we can propose that successive cambia arise from interxylary phloem by topological change of the new cambia differentiation migrating from secondary phloem parenchyma to pericycle (heterotopy), as a homologue of the coalescent cambium, but with a larger extension, so large that they start constituting completely independent additional cambia. Whether these interpretations would hold under a genetic developmental approach is something yet to be explored. These combinations of developmental processes as observed in Nyctaginaceae may be under complex gene regulation, given that multiple cellular and tissue processes are involved in the formation of each cambial variant [32] and that morphological fuzziness results from overlapping developmental programs [57]. In addition, it is likely that the formation of the primary vascular system function as a module independent from the establishment of secondary growth, since different secondary architectures can evolve from distinct pre-vascular conditions in the primary stem.

### The impact of transitions in habits, eustele types and cambial variants in the diversification of Nyctaginaceae

The diversification of Nyctaginaceae was most probably in the Middle Eocene (~ 48 Ma), when most of the extant angiosperm families were already established forming the contemporaneous tropical biomes [45]. Other estimates for the split between Nyctaginaceae and close-related families assumes an interval lying between 13 and 33 Ma [85], as inferred for the divergence from Aizoaceae + Phytolaccaceae (e.g., 26 Myr, [86]).

Speciation/diversification rate increased in Nyctaginaceae 28.62 Ma, at the time of emergence of a group comprising the Bougainvilleeae and Pisonieae (‘‘B&P’’) clade + the Nyctagineae (“NAX”) clade [35]*,* and has been maintained since then. There is not an apparent unique characteristic for this group that could explain its increase in diversification. However, different hypotheses have been pointed out for the high number of species in each tribe individually. For instance, a remarkable radiation of genera from the NAX clade occurred in deserts of North America, and they are associated with multiple evolutions of cleistogamy and edaphic endemism to grow on gypsum soils [35]; the B&P clade stands out by having most of the neotropical and large, woody species of the family, which include both the *Guapira/Neea/Pisonia* trees, as well as the shrubby-scandent or tree species of *Bougainvillea* [35]. In addition, the evolution of fleshy anthocarps (the fruits of Nyctaginaceae) in the *Guapira/Neea* lineage and the likely appearance of endozoochory, seems to be one of the possible explanations for the rapid radiation of taxa of this lineage [35, 36].

*Commicarpus* has experienced a high turnover of species, where many species have been generated but also went extinct, as observed by the rise in both speciation and extinction rate that ultimately involve an increase in the diversification rate. This genus is one of several lineages of Caryophyllales, where a diversification rate shift has been detected, indicating a very recent and rapid radiation [87]. In some other caryophyllalean lineages, genome duplications (polyploidy species) were associated with diversification shifts, which was not identified in *Commicarpus* in that study sampling. Within the NAX clade, *Commicarpus* stands out for having few American species and being mostly diverse in Africa, with several species showing restricted distributions (endemics) in tropical regions, some of them also growing on gypsum or limestone [35, 88, 89].

Our results also suggest that there was no increase in diversification rate in the lineages containing lianas (e.g., *Bougainvillea*, *Colignonia*), therefore, contradicting previous hypotheses that the evolution of the lianescent habit boosts diversification [30, 31]. This observation is noteworthy, because it indicates with an explicit analysis that higher speciation rates correlated to the evolution of lianas seem not to be a rule, at least when the whole group has a cambial variant, as it is the case of Nyctaginaceae. Indeed, while diversification rate has been directly correlated to lianescent habit in Annonaceae [90], not all lianescent lineages have a rise in diversification rate. On the other hand, we found out that there is a transition from regular to polycyclic eustele in the clade comprising the tribes Boldoeae + Colignonieae + Pisonieae + Bougainvillea + Nyctagineae, which is probably the main vascular character associated to an increase in diversification rate (Fig. 11). However, diversification rate shift results obtained with BAMM (Fig. 11) show that a slight increase in diversification rate occurred in a clade comprising Pisonieae + Bougainvillea + Nyctagineae, a less inclusive group compared to Boldoeae + Colignonieae + Pisonieae + Bougainvillea + Nyctagineae observed in the HiSSE results, thus possibly the shift to a polycyclic eustele (medullary bundles) type was a precursor, or a background variable, for diversification rate shift instead of a trigger [91]. This may suggest that other factors, including reproductive traits combined with those analysed here, can be involved in the diversification rate shifts within Nyctaginaceae, such as extrinsic variables, such as habitat occupation. Nevertheless, the occurrence and diversity of variant anatomies in Nyctaginaceae seem to be not contingent on specific habits, since both cambial variants occurs in species with all the range of growth forms present in the family.

## Conclusions

By comparing the stem developments in all main lineages of Nyctaginaceae, we discovered that the mature vascular architectures range from typical successive cambia to interxylary phloem, following four different ontogenies. These ontogenies share developmental stages and thus may contain intermediate forms between the typical state of these two cambial variants. This way, the stem diversity in Nyctaginaceae, which is driven by developmental changes triggered by heterochronic, heterotopic and homeotic processes, may represent a strong case of continuum morphology represented by the evolution of successive cambia from interxylary phloem. Nyctaginaceae is also one of the first groups to show cambial variants in all members of the family, whose ancestor was reconstructed as having interxylary phloem instead of the most endorsed type, successive cambia. These cambial variants are built upon two dissimilar primary vascular organizations, the regular or polycyclic eustele, suggesting that distinct developmental modules are present in the stem ontogeny of these plants. We also presented that high species richness in Nyctaginaceae has probably not been driven by transitions in habits or cambial variants, which indicates that other functional traits, such as the acquisition of medullary bundles, may have been more important in their diversification. Medullary bundles may be advantageous in xerophytic plants, being present in other groups, such as Amaranthaceae and Cactaceae. The complex and diverse developmental pathways shown by Nyctaginaceae may be present in close-related families and be of important phylogenetic significance within Caryophyllales, given the likely potential for convergent evolution in this group. Further investigations in the evolution of development of other caryophyllalean families remain essential in our desire for a better interpretation of the morphological evolution in this lineage. In addition, understanding the genetic regulatory network underlying stem development in Nyctaginaceae seems to be the next step, since it will be easier to identify the role of genes once it is investigated in plants, where the developmental and evolutionary patterns are further comprehended.

## Supplementary Information

**Additional file 1: Table S1.** List of studied species for all Nyctaginaceae and outgroups.

**Additional file 2: Table S2.** Character data set used in HiSSE analyses.

**Additional file 3: Text S1.** R script with parameters for HiSSE analyes.

**Additional file 4: Text S2.** Nexus tree used for diversification analyses.

**Additional file 5: Figure S1.** BAMM results or rate shifts in Nyctaginaceae—all most credible shift sets recovered with associate probabilities.

**Additional file 6: Table S4.** Classification and terminology of successive cambia in different plant families.

## Data Availability

The data set(s) supporting the conclusions of this article are available at https://github.com/ilcneto/Stem_Evolution_Nyctaginaceae. All supporting data are available in Additional files.

## Declarations

### Glossary

**Table:**

**Term**	**Definition**
Cambial variant	Vascular anatomy in mature stems distinct from the so-called ‘regular secondary growth’ (see definition below).
Cambium	Lateral meristem composed of cells actively dividing to maintain themselves, and to produce both secondary xylem and phloem.
Cambium from the continuous cylindrical procambium (CCP)	In polycyclic steles, cambium originated from the outermost ring of vascular bundles.
Coalescent (arching) cambium	In stems with interxylary phloem, fragment of cambium originated through redifferentiation of secondary phloem parenchyma cells encapsulating a portion of phloem and cambium; this (arching) cambium typically connects with the single main cambium at both ends.
Conjunctive tissue	In species with successive cambia, parenchymatous or sclerified tissue between vascular rings derived from the meristematic zones.
Continuous cylindrical procambium (CCP)	In stems with polycyclic eusteles, ring of procambial cells which originates vascular bundles and delimits the pith containing medullary bundles; after differentiation of the bundles, the CCP may be divided into fascicular and interfascicular procambia.
Extra-fascicular cambium	First cambium of some stems which is formed outside the vascular bundles of the eustele in a pericyclic region.
Interxylary phloem	Cambial variant formed through the activity of a single cambium, producing strands of secondary phloem embedded within the secondary xylem.
Medullary bundles	Vascular bundles scattered in the pith, derived from vascular traces of lateral appendages (e.g., leaves, thorns); the bundles usually undergo secondary growth forming secondary axial and radial tissues; in this case they may be named as **vascular fascicule**.
Meristematic zone	In species with successive cambia, the group of cells with meristematic activity produced from divisions of the pericycle (cortex or phloem in other taxa) that originates a new cambium, and conjunctive tissue.
Polycyclic eustele	Eustele formed by medullary bundles scattered in the pith and surrounded by a CCP and their derived vascular bundles.
Regular cambium	Formed from both procambial remnants between primary xylem and phloem (at the vascular bundle) and adjacent (pericyclic) parenchyma. The most common secondary growth mode in woody plants.
Regular cylinder	The main cylinder of secondary xylem and phloem formed by either the cambium from the CCP or the regular cambium; in the latter, it may also be referred as central cylinder.
Regular eustele	Primary vascular system formed by a discrete cylinder of vascular bundles delimiting the pith.
Regular growth	Ancestral secondary growth mode of the Lignophytes, i.e., by means of a bifacial cambium producing secondary xylem inwards and secondary phloem outwards, at homogeneous rate generating a single vascular cylinder.
Sheathing axial parenchyma (SAP)	Axial phloem parenchyma surrounding the interxylary conducting phloem.
Successive cambia	Cambial variant type formed by one or more patches or rings of additional vascular cambia and their products.
Variant cambium	Cambium originated in an unusual position and/or with an activity different from the most common in woody plants. This difference can be in rate, extension, or products. They originate cambial variants.
