# Supplementary material for: Linking the evolution of development of stem vascular system in Nyctaginaceae and its correlation to habit and species diversification
Source: EvoDevo. 2022 Jan 29;13:4. doi: 10.1186/s13227-021-00190-1 (PMC8801151; doi:10.1186/s13227-021-00190-1)
Supplement: Supplementary file 1 — Additional file 1: Table S1. List of studied species for all Nyctaginaceae and outgroups. [file 13227_2021_190_MOESM1_ESM.pdf]

Table S1. Specimen information for stem anatomical analyses of Nyctaginaceae. Names in bold were used for ontogenetic analysis.

Species name, Collector/collector number (Herbarium), Locality/Collection source, Stem diameter. Different collections and their respective information are separated by semicolons.

Taxa	Collector, collector number (Herbarium)[1]	Locality	Habit	Stem diameter (mm)[2,3]
Nyctagineae				
***Abronia fragrans* Nutt. Ex Hook.**	Douglas 2290 (FLAS)	Las Cruces, New Mexico, USA	Herb	4
Abronia neealleyi Standl.	Douglas 2281 (FLAS)	Eddy County, Yeso Hills, New Mexico, USA	Herb	2.5
Acleisanthes acutifolia Standl.	Purpus 4753 (US 842034)*	Coahuila, Mexico	Herb	2
***Acleisanthes chenopodioides* (A.Gray) R.A.Levin.**	Douglas 2289, 2293 (FLAS)	Las Cruces, New Mexico, USA	Herb	3.5
Acleisanthes lanceolata (Wooton) R.A.Levin	Douglas 2277 (FLAS)	Malone Mountains, Sierra Blanca, Texas, USA	Herb	3.5
Acleisanthes longiflora A.Gray.	Douglas 2279 (FLAS)	Malone Mountains, Sierra Blanca, Texas, USA	Herb	2
***Allionia incarnata* L.**	Nee 64124-64126 (USZ); Douglas 2276 (FLAS), 2292	Parque Nacional Amboró, Pampa Grande, Santa Cruz, Bolivia; Malone Mountains, Sierra Blanca, Texas, USA; Luna County, New Mexico, USA	Herb	5
Allionia choisyi Standl.	US 498327	Mesilla Valley, New Mexico, USA	Herb	4
Anulocaulis leiosolenus* (Torr.) Standl. *Var. leiosolenus	Douglas 2278 (FLAS)	Malone Mountains, Sierra Blanca, Texas, USA	Herb	12
Boerhavia diffusa L.	Pace 753 (MEXU)	Veracruz, Mexico	Herb	8
Boerhavia hereroensis Heimerl.	Sukhorukov 517 (MW)	Namib Desert, Karas Region, Namibia	Herb	4
Boerhavia wrightii A.Gray.	Douglas 2288 (FLAS)	Las Cruces, New Mexico, USA	Herb	1.5
***Commicarpus scandens* (L.) Standl.**	Acevedo-Rodríguez 16250 (US); Douglas 2291 (FLAS)	Tonalá, Oaxaca, Mexico; New Mexico, USA	Scandent-shrub	11.5
***Cyphomeris gypsophiloides* (M. Martens & Galeotti) Standl.**	Douglas 2287 (FLAS)	Organ Mountains-Desert Peaks National Monument, Las Cruces, New Mexico, USA	Herb	16

	Mirabilis aggregata (Ortega) Cav.	Pace 728 (MEXU, US)	Ixmiquilpan, Hidalgo, Mexico	Herb	12
	***Mirabilis cf. albida* (Walter) Heimerl.**	Douglas 2286 (FLAS)	New Mexico, USA	Herb	4
	***Mirabilis jalapa* L.**	Acevedo-Rodríguez 16480 (US)	Veracruz, Mexico	Herb	11
	***Nyctaginia capitata* Choisy.**	Douglas 2282 (FLAS)	New Mexico, USA	Herb	5
	***Okenia hypogea* Schltdl. & Cham.**	Pace 749 (MEXU, SPF, US)	Veracruz, Mexico	Herb	5
Pisonieae					
	***Grajalesia fasciculata* (Standl.) Miranda.**	Pace 765 (MEXU, SPF, US)	Chiapas, Mexico	Scandent-tree	12; trunk
	Guapira bracei Britton	Scott 386 (USw 23138)*	Florida, United States of America	Tree	Trunk
	***Guapira pernambucensis* (Casar.) Lundell.**	Cunha Neto 04-05 (HURB)	Alagoinhas, Bahia	Scandent-shrub	23
	***Guapira graciliflora* (Mart. Ex J.A.Schmidt) Lundell.**	Cunha Neto 06 (HURB)	Alagoinhas, Bahia	Tree	22
	Guapira laxa (Netto) Furlan.	Cunha Neto 08-09 (HURB)	Universidade Estadual de Feira de Santana, Feira de Santana, Bahia	Tree	15; trunk
	Guapira linearibracteata	Gentle 481 (USw 29941)*	Belize	Tree	trunk
	Neea amplifolia	Pace 720 (US)	Reserva Biológica La Selva, Sarapiquí, Heredia, Costa Rica	Tree	12; trunk
	Neea delicatula Standl.	Pace 689 (US)	Reserva Biológica La Selva, Sarapiquí, Heredia, Costa Rica	Tree	15
	***Neea hermafrodita* S. Moore**	Nee 64112-64113 (USZ)	Jardín Botánico Municipal de Santa Cruz de la Sierra, Santa Cruz de la Sierra, Bolivia	Tree	11.5
	Neea laetevirens Standl.	Pace 713, 716 (US); Stern 196 (USw 16154)*	Reserva Biológica La Selva, Sarapiquí, Heredia, Costa Rica; Los Santos, Panamá.	Tree	15; trunk
	Neea ovalifolia Spruce ex J.A. Schmidt	Feuillet 10218 (USw 42783)*	Régina, French Guiana	Tree	18.5
	Neea psychotrioides Donn. Sm.	Pace 763 (MEXU)	Estación de Biología Tropical Los Tuxtlas, Veracruz, Mexico.	Tree	9
	***Pisonia aculeata* L.**	Acevedo-Rodríguez 16549 (US)	Bonito, Mato Grosso do Sul, Brazil	Liana	23.5
	Pisonia fragrans Dum.Cours.	Stern, 2456 (USw 35502)*	Dominican Republic	Tree	Trunk

Pisonia ligustrifolia Heimerl	Abbott 2251 (USw 1982)*	Hispaniola Island, Dominican Republic	Tree	21
Pisonia zapallo Griseb.	Nee 64110 (USZ)	Jardín Botánico Municipal de Santa Cruz de la Sierra, Santa Cruz de la Sierra, Bolivia	Tree	10
***Pisoniella arborescens* (Lag. & Rodr.) Standl.**	Pace 738-739 (MEXU, SPF, US)	Alfajayucan, Hidalgo, Mexico	Liana	15.5
***Pisoniella glabrata* (Heimerl) Standl.**	Nee 64137, 64151 (USZ)	Parque Nacional Amboró, Vallegrande, Santa Cruz, Bolivia	Scandent-shrub, liana	11
Bougainvilleeae				
***Belemia fucsioides* Pires.**	Farney 4887, 4888 (RB); Cunha Neto 16 (SPF), 17	Pedro Canário, Espírito Santo, Brazil	Liana	5
***Bougainvillea berberidifolia* Heimerl.**	Nee 64140 (USZ)	Parque Nacional Amboró, Comarapa, Santa Cruz, Bolivia	Shrub	18
Bougainvillea campanulata Heimerl.	Acevedo-Rodríguez 16772 (US); Nee 64142 (USZ)	Mato Grosso do Sul, Brazil; Parque Nacional Amboró, Comarapa, Santa Cruz, Bolivia	Shrub	22
***Bougainvillea modesta* Heimerl.**	Nee 64115 (USZ)	Jardín Botánico Municipal de Santa Cruz de la Sierra, Bolivia	Tree	20
***Bougainvillea stipitata* Griseb.**	Nee 64121 (USZ)	Parque Nacional Amboró, Samaipata, Santa Cruz, Bolivia	Tree	19
Bougainvillea spectabilis Willd.	Rossetto 453 (RB)	Estrada Carlos Chagas-Teófilo Otoni, Minas Gerais, Brazil	Shrub	14
Phaeoptilum spinosum Radlk.	Dechamps 1213 (MADw 37340)*	Mocamedes, Angola	Shrub	20
Colignonieae				
***Colignonia glomerata* Griseb.**	Nee 64157-64159 (USZ)	Parque Nacional Amboró, Samaipata, Santa Cruz, Bolivia	Liana	17
Colignonia rufopilosa Kuntze.	Nee 64061 (USZ)	Cochabamba, Bolivia	Liana	11
Boldoeae				
Cryptocarpus pyriformis Kunth	Fosberg 44705 (US 2833648)*	Galápagos	Liana	5.5
Salpianthus macrodontus Standl.	Annetta 3251 (US 2219249)*	Sinaloa, Mexico	Herb	6
***Salpianthus purpurascens* (Cav. Ex Lag.) Hook. & Arn.**	Pace 774 (MEXU, SPF, US)	El Cobanal, Chiapas, Mexico	Shrub	20

Salpianthus arenarius Humb. & Bonpl.	Hinton 10215 (US 1893480)*	Vallecitos, Guerrero, Mexico	Shrub	3
Leucastereae				
***Andradea floribunda* Allemão**	Rossetto 445 (RB); Serviço Florestal 152 (SPFw 5033)*	Linhares, Espírito Santo, Brazil; Distrito Federal, Brazil.	Tree	21; trunk
***Leucaster caniflorus* (Mart.) Choisy**	Rossetto 447, 455 (RB)	Linhares, Espírito Santo, Brazil; Teófilo Otoni, Minas Gerais, Brazil	Liana	19
***Ramisia brasiliensis* Oliv.**	Rossetto 448 (RB); Serviço Florestal 2614 (SPFw 5035)*	Nanuque, Minas Gerais, Brazil; Minas Gerais, Brazil.	Tree	18; trunk
***Reichenbachia hirsuta* Spreng.**	Nee 64109, 64169 (USZ)	Jardín Botánico Municipal de Santa Cruz de la Sierra, Santa Cruz de la Sierra, Bolivia; Rio Grande, Santa Cruz de la Sierra, Bolivia	Shrub	13
Reichenbachia colombiana Stand.	Dugand 979 (SJRw 32385)*	Colombia	Shrub	35

[1] In this list, specimens with asterisk (*) were obtained in the herbarium or wood collection indicated in parenthesis; the collection number is also indicated.

[2] Largest stem sample for one of the adult specimens analyzed for the anatomical study.

[3] The indication “trunk” means that a sample was obtained from the main stem at ca. 1.30 cm height.

Collections acronyms: FLAS, Florida Museum of Natural History; HURB, Universidade Federal do Recôncavo da Bahia; MADw (Forest Products Laboratory, Madison, WI, USA); MEXU, Universidad Nacional Autónoma de México; RB, Jardim Botânico do Rio de Janeiro; SJRw, Samuel J. Record (acquired by the Forest Products Laboratory, Madison, WI, USA) SPF and SPFw, Universidade de São Paulo; US, Smithsonian Institution; USZ, Museo de Historia Natural Noel Kempff Mercado, Universidad Autónoma Gabriel René Moreno.
