# Supplementary material for: Linking the evolution of development of stem vascular system in Nyctaginaceae and its correlation to habit and species diversification
Source: EvoDevo. 2022 Jan 29;13:4. doi: 10.1186/s13227-021-00190-1 (PMC8801151; doi:10.1186/s13227-021-00190-1)
Supplement: Supplementary file 2 — Additional file 2: Table S2. Character data set used in HiSSE analyses. [file 13227_2021_190_MOESM2_ESM.pdf]

Table S2. Character dataset for HiSSE analyses.

Taxon	habit	secondary_growth	eustele_type
Abronia_ameliae	0	1	1
Abronia_angustifolia	0	1	1
Abronia_bigelovii	0	1	1
Abronia_elliptica	0	1	1
Abronia_fragrans	0	1	1
Abronia_latifolia	0	1	1
Abronia_maritima	0	1	1
Abronia_nana	0	1	1
Acleisanthes_acutifolia	0	1	1
Acleisanthes_angustifolia	0	1	1
Acleisanthes_chenopodioides	0	1	1
Acleisanthes_crassifolia	0	1	1
Acleisanthes_diffusa	0	1	1
Acleisanthes_longiflora	0	1	1
Acleisanthes_purpusiana	0	1	1
Acleisanthes_somalensis	0	1	1
Agdestis_clematidea	1	1	0
Allionia_choisyi	0	1	1
Andradea_floribunda	0	1	0
Anulocaulis_eriosolenus	0	1	1
Anulocaulis_leiosolenus_var_gypsogenus	0	1	1
Anulocaulis_leiosolenus_var_leiosolenus	0	1	1
Boerhavia_coccinea	0	1	1
Boerhavia_cordobensis	0	1	1
Boerhavia_coulteri_var_palmeri	0	1	1
Boerhavia_diffusa	0	1	1
Boerhavia_gracillima	0	1	1
Boerhavia_hereroensis	0	1	1
Boerhavia_linearifolia	0	1	1
Boerhavia_purpurascens	0	1	1
Boerhavia_spicata	0	1	1
Boerhavia_triquetra_var_intermedia	0	1	1
Bougainvillea_spectabilis	1	1	1
Bougainvillea_campanulata	0	1	1
Bougainvillea_infesta	0	1	1
Bougainvillea_spinosa	0	1	1
Bougainvillea_stipitata	0	1	1
Colignonia_glomerata	1	1	1
Colignonia_parviflora_subsp_biumbellata	1	1	1
Colignonia_scandens	1	1	1
Commicarpus_arabicus	0	1	1

Commicarpus_australis	1	1	1
Commicarpus_boissieri	1	1	1
Commicarpus_commersonii	1	1	1
Commicarpus_fallacissimus	1	1	1
Commicarpus_grandiflorus	1	1	1
Commicarpus_helenae	1	1	1
Commicarpus_mistus	0	1	1
Commicarpus_pedunculosus	0	1	1
Commicarpus_pentandrus	1	1	1
Commicarpus_plumbagineus	1	1	1
Commicarpus_scandens	1	1	1
Commicarpus_sinuatus	1	1	1
Commicarpus_stenocarpus	0	1	1
Commicarpus_tuberosus	0	1	1
Cryptocarpus_pyriformis	1	1	1
Cuscatlania_vulcanicola	0	1	1
Cyphomeris_crassifolia	0	1	1
Cyphomeris_gypsophiloides	0	1	1
Gallesia_integrifolia	0	1	0
Gisekia_pharnacioides	0	0	0
Grajalesia_fasciculata	1	1	1
Guapira_discolor	0	1	1
Guapira_eggersiana	0	1	1
Guapira_obtusata	0	1	1
Hilleria_latifolia	0	0	0
Leucaster_caniflorus	1	1	0
Mirabilis_albida	0	1	1
Mirabilis_exserta	0	1	1
Mirabilis_himalaica	0	1	1
Mirabilis_jalapa	0	1	1
Mirabilis_linearis	0	1	1
Mirabilis_multiflora	0	1	1
Mirabilis_nyctaginea	0	1	1
Mirabilis_oxybaphoides	0	1	1
Mirabilis_pringlei	0	1	1
Mirabilis_violacea	0	1	1
Neea_belizensis	0	1	1
Neea_psychotrioides	0	1	1
Nyctaginia_capitata	0	1	1
Okenia_hypogaea	0	1	1
Petiveria_alliacea	0	1	0
Phaeoptilum_spinosum	0	1	1
Phytolacca_americana	0	1	0
Phytolacca_dioica	0	1	1

Pisonia_aculeata	1	1	1
Pisonia_brunoniana	0	1	1
Pisonia_macranthocarpa	0	1	1
Pisonia_rotundata	0	1	1
Pisonia_subcordata	0	1	1
Pisonia_silvatica	0	1	1
Pisonia_umbellifera	0	1	1
Pisonia_zapallo	0	1	1
Pisoniella_arborescens	1	1	1
Ramisia_brasiliensis	0	1	0
Reichenbachia_hirsuta	0	1	0
Rivina_humilis	0	1	0
Salpianthus_arenarius	0	1	1
Salpianthus_macrodontus	0	1	1
Seguieria_aculeata	1	1	0
Trichostigma_octandrum	1	0	0
Tripterocalyx_carneus	0	?	1
Tripterocalyx_crux_maltae	0	?	1
Tripterocalyx_micranthus	0	?	1
