# Supplementary figures and images for: Linking the evolution of development of stem vascular system in Nyctaginaceae and its correlation to habit and species diversification
Source: EvoDevo. 2022 Jan 29;13:4. doi: 10.1186/s13227-021-00190-1 (PMC8801151; doi:10.1186/s13227-021-00190-1)

$f = 0.044$

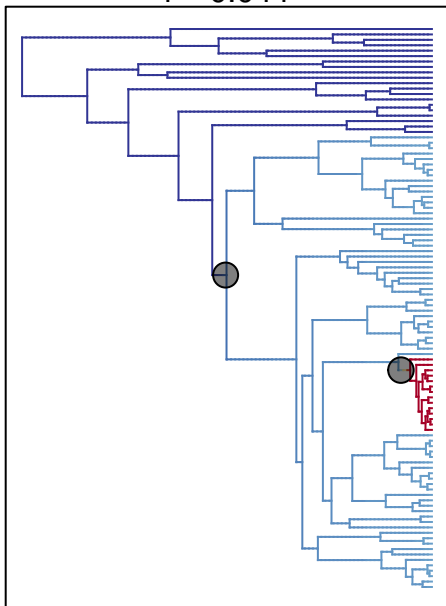

$f = 0.039$

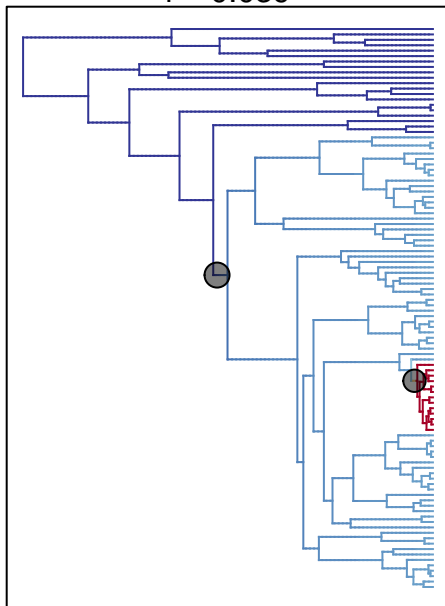

$f = 0.03$

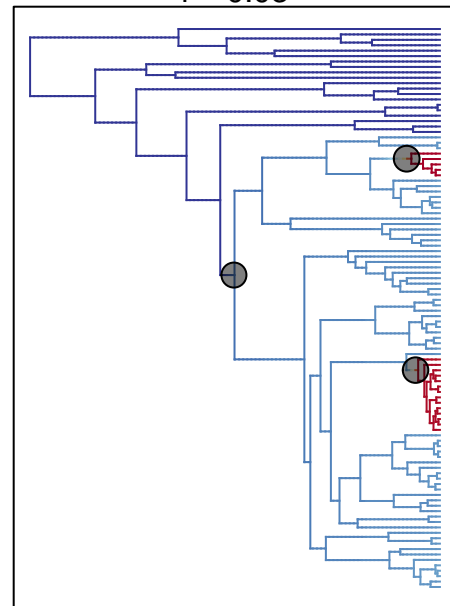

$f = 0.026$

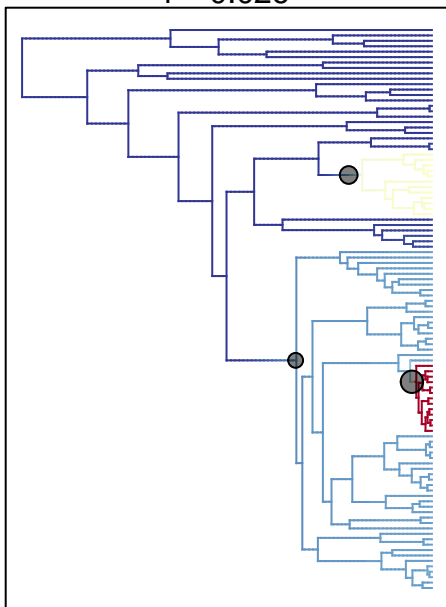

$f = 0.025$

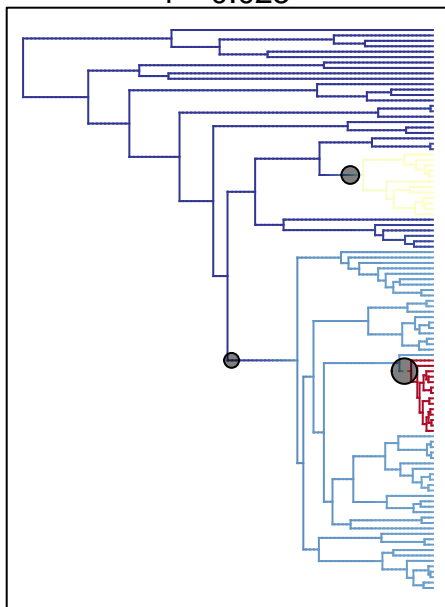

$f = 0.025$

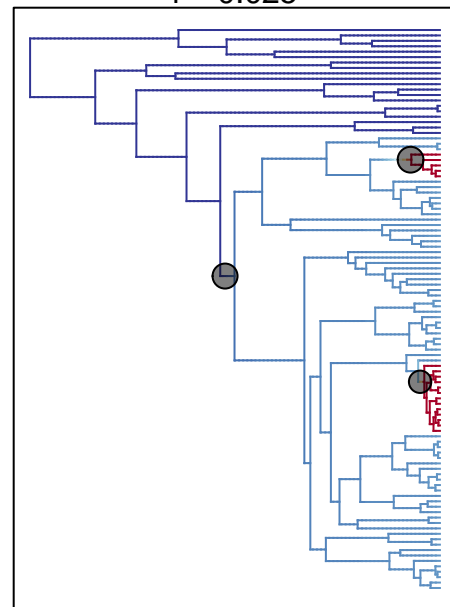

$f = 0.022$

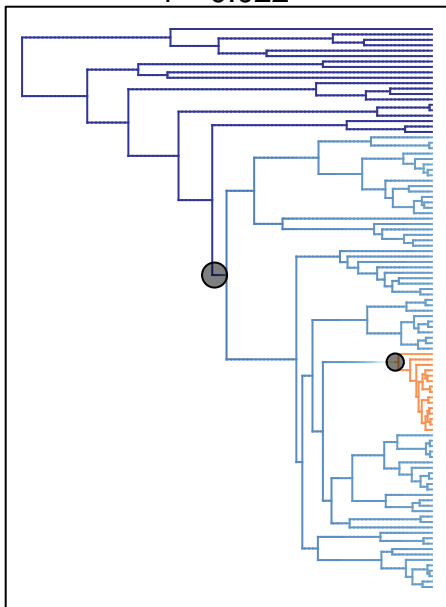

$f = 0.02$

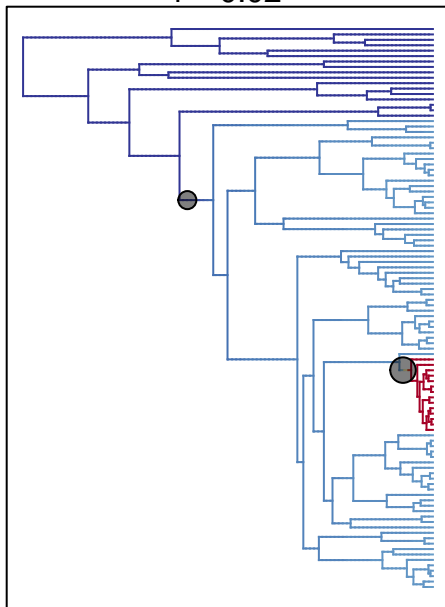

$f = 0.02$

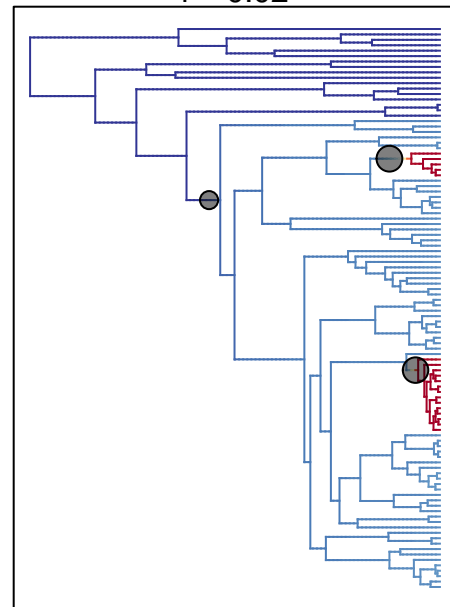

Supplement: Supplementary file 5 — Additional file 5: Figure S1. BAMM results or rate shifts in Nyctaginaceae—all most credible shift sets recovered with associate probabilities. [file 13227_2021_190_MOESM5_ESM.pdf]
