# Supplementary material for: Linking the evolution of development of stem vascular system in Nyctaginaceae and its correlation to habit and species diversification
Source: EvoDevo. 2022 Jan 29;13:4. doi: 10.1186/s13227-021-00190-1 (PMC8801151; doi:10.1186/s13227-021-00190-1)
Supplement: Supplementary file 6 — Additional file 6: Table S4. Classification and terminology of successive cambia in different plant families. [file 13227_2021_190_MOESM6_ESM.pdf]

Table S4. Classification and terminologies applied to successive cambia in different plant families.

**Author**	**Terminology**	**Author's description**	**Taxa**
Carlquist (2001) [1]	*Atriplex* type	Secondary growth by vascular cambia produces abundant secondary xylem.	*Atriplex*, Chenopodiaceae
	Pisonia type	Vascular cambia produce limited strands of secondary xylem.	*Pisonia,* Nyctaginaceae
	Securidaca type	Phloem as a band rather than as strands.	*Securidaca,* Polygalaceae
Schweingruber et al. (2011) [2]	Caryophyllaceae type	Large irregular bands of unlignified parenchyma and phloem cells within the stem.	Caryophyllaceae
	Concentrically arranged single vascular bundles	"Vascular bundles", consisting of xylem and phloem, are separated by parenchyma cells.	Amaranthaceae
	Concentric continuous successive cambia	The successive cambia produce tangential bands of lignified xylem and radial strips of unlignified parenchyma and phloem.	Aizoaceae, Amaranthaceae
	Diffuse = foraminate	More or less irregularly arranged "vascular bundles" are located in a conjunctive tissue.	Amaranthaceae
Cunha Neto et al. (2018) [3]	Continuous successive cambia	When a new cambium and its derivatives form a complete ring around the regular vascular cylinder;	*Serjania pernambucens*is, Sapindaceae
	Discontinuous successive cambia	When a new cambium and its derivatives form eccentric arcs around the regular vascular cylinder (i.e., they are not complete rings).	Species of *Paullinia*, Sapindaceae
Zumaya-Mendoza et al. (2019) [4]	Successive cambia in concentric rings	The additional secondary xylem and conjunctive tissue are formed in continuous bands.	Species of *Iresine,* Amaranthaceae
	Successive cambia in patches	The new tissue is formed in patches of phloem and conjunctive tissue.	Species of *Iresine,* Amaranthaceae

References

1. Carlquist S. Comparative wood anatomy. Systematic, ecological and evolutionary aspects of dicotyledon wood. 2nd ed. Berlin: Springer Verlag; 2001.

2. Schweingruber FH, Börner A, Schulze E-D. Atlas of Stem Anatomy in Herbs, Shrubs and Trees. Berlin: Springer-Verlag; 2011.

3. Cunha Neto IL, Martins FM, Somner GV, Tamaio N. Successive cambia in liana stems of Paullinieae and their evolutionary significance in Sapindaceae. Bot J Linn Soc. 2018;186:66–88.

4. Zumaya-Mendoza S, Aguilar-Rodríguez S, Yáñez-Espinosa L, Terrazas T. Stem anatomy diversity in Iresine (Amaranthaceae s.l.): an ecological interpretation. Rev Bras Bot [Internet]. Springer International Publishing; 2019;42:329–44. Available from: https://doi.org/10.1007/s40415-019-00530-5
